# Differential Effects of DHA-Rich Fish Oil Supplementation on Intestinal and Pulmonary Alterations in Experimental Malaria Caused by *Plasmodium berghei* NK65

**DOI:** 10.1021/acsomega.5c12964

**Published:** 2026-03-20

**Authors:** Ludmila Ponce Monken Custódio Pereira, José Henrique Silva-Rodrigues, Fernanda Martins Pinheiro, Letícia Ferreira Machado, Ana Carolina Fonseca da Silva, Carolina Brandi Marques, Milena Tavares Gomes, Fernanda Mikaela Moreira Gonçalves, Jéssica Correia Bezerra-Bellei, Gabriel Brum, Vanessa Cordeiro Dias, João Renato Hipólito, Andre Netto Bastos, Haroldo Lobo dos Santos Nascimento, Flávia Marcia de Castro e Silva, Heloisa D’Avila, Adolfo Firmino Neto, Janildo Ludolf Reis Junior, Vinicius Novaes Rocha, Flávia Lima Ribeiro-Gomes, Juciane Maria de Andrade Castro, Kézia Katiani Gorza Scopel

**Affiliations:** † Research Centre of Parasitology, Department of Parasitology, Microbiology and Immunology and Post-Graduate Program in Biological Science, 28113 Federal University of Juiz de Fora, Juiz de Fora 36036-900, Minas Gerais, Brazil; ‡ Reproductive Biology Centre, Federal University of Juiz de Fora, Juiz de Fora 36036-900, Minas Gerais, Brazil; § Cell Biology Laboratory, Department of Biology, Federal University of Juiz de Fora, Juiz de Fora 36036-900, Minas Gerais, Brazil; ∥ Cortes Villela Laboratory, Juiz de Fora 36020-200, Minas Gerais, Brazil; ⊥ Laboratory of Histology, Department of Morphology, Federal University of Juiz de Fora, Juiz de Fora 36036-900, Minas Gerais, Brazil; # Research Centre of Pathology and Veterinary Histology, Department of Veterinary Medicine, Federal University of Juiz de Fora, Juiz de Fora 36036-900, Minas Gerais, Brazil; ∇ Department of Parasitology, Microbiology and Immunology, Federal University of Estado do Rio de Janeiro, Rio de Janeiro 20550-270, Rio de Janeiro, Brazil; ○ Laboratory of Malaria Research, Oswaldo Cruz Institute/Fiocruz, Rio de Janeiro 21040-360, Rio de Janeiro, Brazil

## Abstract

Malaria infection can trigger Acute Respiratory Distress Syndrome (ARDS), a potentially lethal complication resulting, among other factors, from an exacerbated inflammatory response associated with the infection. In addition, the disease appears to impact the intestinal tract, causing disorders that in turn can influence the severity of lung damage due to bidirectional communication between these organs, known as the gut-lung axis. DHA-rich fish oil (omega-3) has been demonstrated to mitigate lung inflammation in mice infected with *Plasmodium berghei* ANKA, a highly virulent parasite strain. However, it remains unclear whether this effect extends to any species or strain of *Plasmodium*. Therefore, in this study, the potential of DHA-rich fish oil to modulate lung injury was evaluated during the experimental infection of C57BL/6 mice infected with *P. berghei* NK65. In addition, we also analyzed the effect of supplementation on the intestinal environment. Although DHA-rich fish oil supplementation was associated with partial preservation of intestinal morphology, including reduced intestinal shortening, these effects did not translate into improved pulmonary outcomes. These findings highlight the strain- and pathogenesis-dependent effects of ω-3 supplementation, emphasizing distinct responses across different infection models. Specifically, our results reinforce the importance of considering the diversity of pathogenic profiles among *Plasmodium* strains in the investigation of nutritional or immunomodulatory interventions.

## Graphical abstract

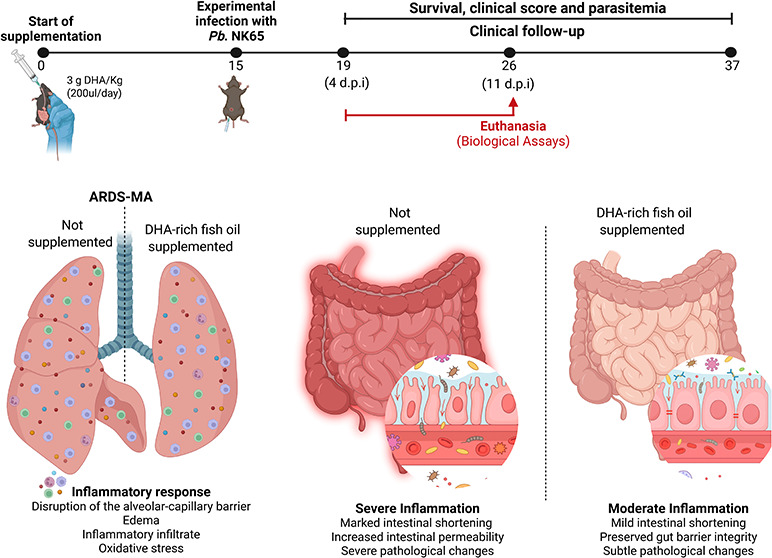

## Introduction

Among the main complications related to malaria infection, a parasitic disease responsible for more than 600,000 deaths annually,^1^ is acute respiratory distress syndrome (MA-ARDS).^2^ In contrast to cerebral malaria (CM), which occurs mainly in *Plasmodium falciparum* infections, MA-ARDS can be triggered during infection by any *Plasmodium* species, although it is more prevalent in *P. falciparum* and *Plasmodium vivax* infections (reviewed by Claser et al.,).^3^ It is a highly lethal pathology in adults (50–80% of deaths) when compared to children and can occur during or after adequate antimalarial treatment.^4–6^

The factors that trigger pulmonary pathology in malaria infections are not yet well understood. However, studies with murine models suggest that antigens and Pathogen-Associated Molecular Patterns (PAMPs) (such as hemozoin) released during the erythrocytic cycle of the parasites, as well as the adhesion of parasitized red blood cells/leukocytes to the pulmonary vascular endothelium, are important in this process.^7–10^ During malaria infection, monocytes that remain in the tissue as macrophages are activated by hemozoin and/or other substances derived from the parasite via the inflammasome, which leads to M1 polarization, resulting in the production of pro-inflammatory cytokines and oxidative metabolites that contribute to lung injury.^3,11,12^ The persistence of inflammation induces the recruitment of T lymphocytes, a process largely mediated by chemokines and their specific receptors.^13^ The interaction between these mediators and their receptors promotes the adhesion of T cells to the endothelium and their extravasation into the lung parenchyma, where they amplify the inflammatory response through pro-inflammatory cytokines, aggravating the condition.^14–16^

It is currently believed that changes in the gut microbiota and the production of microbial metabolites can modulate the systemic inflammatory response, which may influence the severity of lung injury. This occurs due to the bidirectional communication pathway between the gastrointestinal tract and the lungs (called the gut-lung axis), mediated by immunological, microbial, and metabolic factors.^17^ MA-ARDS is characterized by a breakdown of the capillary-alveolar barrier, resulting in interstitial and alveolar edema, microhemorrhages, and intense inflammatory infiltrate into the alveoli.^7^ To date, there is no specific treatment available for MA-ARDS, and patients with this syndrome are managed with conventional therapies applied to nonmalarial ARDS.

Polyunsaturated fatty acids (PUFAs) of the omega-3 (ω-3) family, such as eicosapentaenoic acid (EPA) and docosahexaenoic acid (DHA), are macronutrients that have important anti-inflammatory effects. The anti-inflammatory responses of these fatty acids involve several mechanisms, including modifying the composition of fatty acids in cell membrane phospholipids, giving rise to anti-inflammatory and proresolving mediators such as resolvins, protectins, and maresins, which also promote the rupture of lipid rafts and inhibit the activation of nuclear factor κB (NF-κB), an important pro-inflammatory transcription factor. As a result, the expression of genes linked to inflammatory pathways (such as NF-κB) is downregulated, in addition to the activation of the peroxisome proliferator-activated receptor γ (PPAR-γ), a transcription factor with anti-inflammatory properties.^18^ In this context, it has been suggested the protective potential of ω-3 fatty acids in the treatment of cancer patients, including lung cancer, due to their ability to inhibit disease progression or reduce complications.^19^ In addition, it has been shown that enteral administration of a nutritional formula containing PUFAs in patients with ARDS was able to significantly improve oxygenation, ventilation, and lung compliance.^20^

In parasitic diseases, such as schistosomiasis, leishmaniasis, and malaria, among others, dietary supplementation with ω-3 PUFAs resulted in low parasitism, inflammatory profile, and oxidative stress (reviewed by Alhusseingy and El-Beshbishi).^21^ Specifically in malaria, in vitro studies have showed an association between DHA and reduced parasitism, particularly in mature parasite stages and gametocytes,^22,23^ whereas previous studies conducted by our group has demonstrated that C57BL/6 mice supplemented with DHA-rich fish oil become resistant to cerebral and pulmonary malaria induced by experimental infection with *P. berghei* ANKA (PbA).^24,25^ However, an important knowledge gap remains regarding host–parasite interactions, specifically whether the immunomodulatory properties of ω-3, especially with high DHA concentration, can be extrapolated between different strains and species of parasites. Thus, the present study proposes to evaluate the effect of supplementation with DHA-rich fish oil on protection against lung damage caused by experimental infection with *P. berghei* NK65 (PbN). Additionally, the impact of infection on the intestinal environment of supplemented and nonsupplemented animals was also investigated. PbA and PbN parasites promote distinct clinical pictures during the course of infection: PbA causes neurological dysfunction (named cerebral malaria), while PbN leads to lung injury and severe anemia.

## Materials and Methods

### Mice, Supplementation, and Infection

Female C57BL/6 mice, aged between 6 and 8 weeks, were obtained from the Reproductive Biology Center at the Federal University of Juiz de Fora/UFJF, Minas Gerais, Brazil. The animals were kept in the animal facility of the Parasitology Research Center (NUPEP), housed in polypropylene cages placed on ventilated shelves with regulated temperature and light exposure (12 h light/12 h dark) with filtered water and feed (Nuvitall) *ad libitum*, in addition to daily veterinary monitoring to verify their welfare conditions.

The animals were supplemented with DHA-rich fish oil (per capsule: 500 mg of DHA, 150 mg of EPA [3:1 DHA:EPA]; Essential Nutrition; see Table S1 for more nutritional information). Each animal received a daily oral dose of 3 g DHA/kg body weight by gavage, starting prior to infection.^24^ The selection of this dose was based on our previous studies in which we demonstrated that the effects of DHA-rich fish oil supplementation were dose-dependent. Specifically, only 20% of animals were protected from neurological symptoms at a dose of 1.5 g DHA/kg, whereas 60% of animals were protected at 3 g DHA/kg.^25^ Similarly, pulmonary injury was also mitigated at the 3 g DHA/kg dose.^24^

Fifteen days after the start of enteral supplementation, the animals were infected with 10^5^ infected red blood cells (iRBCs) with PbN, intraperitoneally (i.p.). The supplementation was maintained during all of the time of experimentation.

### Clinical Evaluation, Survival, Parasitemia, and Hematological Analysis

After experimental infection, the animals were monitored to determine the clinical evolution (score, survival, and evolution of parasitemia) of the infection in animals supplemented or not supplemented with oil. Daily clinical evaluation was initiated on the fourth d.p.i. (day postinfection) and maintained until death as previously described.^26^ Parameters such as spontaneous activity (SA), limb grasping (LG), body tone (BT), trunk curvature (TC), piloerection (PE), tremors (Sh), abnormal breathing (AB), dehydration (D), incontinence (I), and paralysis (P) were assessed daily to determine the clinical disease severity score. These parameters were scored as 0 (absent) or 1 (present) for TC, PE, Sh, and AB, and 0 (normal), 1 (intermediate), or 2 (severe) for the others. The total clinical score was calculated using the following formula: SA + LG + BT + TC + PE + Sh + AB + 3 × (D + I + P). Deaths observed during follow-up were recorded and used to construct the survival curve.

Starting on the fourth day of the i.p., to quantify the peripheral parasitemia, an incision was made at the tip of the tail to obtain a drop of blood/mouse, which was spread on the slide and stained with Giemsa. The smears were then analyzed under an optical microscope (1000× magnification).^24^

For hematological analyses (total leukocytes, erythrocytes, platelets, hematocrit, and hemoglobin), 500 μL of blood from each animal was stored in collection tubes containing EDTA (Labor Import). The analyses were performed automatically on a HEMIX 5-60 device.

### Analysis of Alveolar Vascular Integrity

Evans blue staining was used to assess pulmonary vascular integrity, since the dye binds to serum albumin as a vehicle for circulation in the bloodstream, crossing the vascular endothelium only in the case of injury. The protocol used was that previously described.^24^ On the 11th day of i.p., the animals were anesthetized with inhaled isoflurane (Syntech) and then received 200 μL of 2% Evans Blue solution (Sigma-Aldrich) intravenously via the orbital plexus. After 45 min, euthanasia was performed, followed by cardiac perfusion with 10 mL of 1× phosphate-buffered saline (PBS). The lungs were then obtained and immersed in 2 mL of a 10% formamide solution (Sigma-Aldrich) to recover the extravasated dye. After 48 h of incubation at 37 °C, the reading was performed with a spectrophotometer at 620 nm (Varioskan; ThermoFisher Scientific). The concentration of the extravasated dye was determined from a serial dilution standard curve, starting from a concentration of 50 μg/mL.

The occurrence of pulmonary edema was determined as previously described.^26^ For this purpose, after euthanasia, the nonperfused lungs were removed from the rib cage, washed with 1× PBS, dried with paper towels, and weighed on an analytical balance (Bioscale).

### Bronchoalveolar Lavage (BAL) for the Evaluation of Cell Profile, Protein Concentration, and Oxidative Stress

After euthanasia, the trachea of the mice was exposed and cannulated with a sterile catheter, followed by instillation and aspiration of 1 mL of 1× PBS into the lungs. The aspirate (approximately 0.8 mL) was centrifuged at 700*g* for 10 min. The supernatant was stored at −80 °C and then used to determine protein concentration using the BCA Protein Assay Kit (ThermoFisher Scientific).^27^ Next, the total number of cells in the sediment was determined by counting them in a Neubauer chamber. For differential counting (macrophages, lymphocytes, and neutrophils), slides containing 4 × 10^4^ cells/mL were prepared using the Cytospin centrifuge (Cytospin, ThermoFisher Scientific). After staining with panoptic (Laborclin), differential cell counting (macrophages, lymphocytes, and neutrophils) was performed using optical microscopy.

Oxidative stress was analyzed by measuring the H_2_O_2_ (hydrogen peroxide) release by BAL cells. For this purpose, 2 × 10^5^ cells/mL were resuspended in 1 mL of phenol red solution (10× PBS; 5.5 mM dextrose; 0.56 mM phenol red; Sigma-Aldrich); 8.5 u/mL HRP type II (Sigma-Aldrich). Next, 100 μL of cell suspension was plated in triplicate in 96-well plates and incubated for 1 h at 37 °C. PMA (Phorbol Myristate Acetate −1 μg/mL, Sigma-Aldrich), an agent capable of inducing the release of H_2_O_2_ by inflammatory cells, was used as a positive control. The reaction was stopped by adding 10 μL of NaOH/well, and the reading was performed on a spectrophotometer (Agilent BioTek Epoch) at 620 nm. The conversion of absorbance to μM of H_2_O_2_ was performed by comparison with the standard curve of known concentrations of H_2_O_2_ (5–40 μM).^22^ The tests were performed in triplicate.

### Histopathology

For histopathological analysis, after euthanasia followed by cardiac perfusion, fragments of lung and intestine (small and large intestine samples) were fixed in 10% formaldehyde, processed following routine histological procedures, and embedded in paraffin. Then, sections (5 μm) were stained with hematoxylin and eosin and analyzed under a light microscope equipped with a camera (Olympus BX53).

### Isolation and Phenotyping of Lung Cells

To characterize the pulmonary cell populations, the organ of each animal was collected, washed with 1× PBS, and cut into pieces of approximately 1 mm with sterile scissors, followed by incubation with 1 mL of digestion solution (RPMI without FBS, 0.5 mg/mL DNase I, and 1 mg/mL collagenase I) at 37 °C for 30 min. After this process, 500 μL of RPMI medium supplemented with 5% fetal bovine serum (FBS) was used to stop tissue digestion. The tissue was then macerated in a cell strainer (70 μm, BD Biosciences). The cell suspensions were centrifuged at 500*g* for 10 min at 4 °C, and the sediment was resuspended in complete RPMI 1640 medium (5% FBS) for subsequent staining.

For immunophenotyping, the cell suspension was transferred to 96-well plates (V-bottom) and centrifuged at 500*g* for 10 min at 10 °C. The supernatants were then discarded, and the cells were resuspended in 50 μL of cell viability marker (LIVE/DEAD Fixable Violet Dead Cell Stain Kit, Invitrogen) with dilutions according to the manufacturer’s recommendations and incubated for 30 min at 4 °C. The cells were then washed with FACS buffer (1× PBS with 5% FBS) and centrifuged again at 500*g* for 10 min at 4 °C. The cells were then incubated for 30 min at 4 °C with 50 μL of FACS buffer containing the following antibodies: antimouse TCR PE (H57–597, BD Pharmingen), antimouse CD45 FITC (30F11, BD Pharmingen), antimouse CD11b PerCP-Cy5.5 (M1/70, BD Pharmingen), antimouse CD4 APC-H7 (GH1.5, BD Pharmingen), or antimouse CD8 AF700 (53-6.7; BD Pharmingen). Subsequently, unbound antibodies were removed by washing, and the cells were resuspended in 200 μL of PBS and analyzed by flow cytometry.

Fluorescence-minus-one (FMO) controls were used to establish the analysis gates. In the gate strategy (Figure S1A), the region was defined based on the forward scatter (FSC) and side scatter (SSC) profiles. Dead cells were removed using live/dead staining, and single cells were identified on an FSC-H vs FSC-A scatter plot, ensuring the exclusion of cell aggregates. Next, the cell populations of interest were defined as described: total leukocytes: CD45+; myeloid cells: CD45+CD11b+; total T lymphocytes: CD45+/CD11b-/TCR+; CD4 T lymphocytes: CD11b-/TCR+/CD4+; CD8 T lymphocytes: CD11b-/TCR+/CD8+; inflammatory monocytes: CD11b+/Ly6Chi/Ly6-. Approximately 1,000,000 events per sample were acquired using a BD FACSCelesta, and the data were analyzed using FlowJo software (version 10.0).

### Cytokine Assay

After euthanasia, the left lung lobe of each animal was stored in microtubes and kept at −80 °C until processing to obtain tissue homogenate. For this purpose, the lung tissue was macerated in extraction solution (0.4 M NaCl, 0.05% Tween-20, 0.5% BSA-bovine serum albumin, 0.1 mM phenylmethylsulfonyl fluoride, 0.1 mM benzethonium chloride, 10 mM EDTA, 0.02% aprotinin), using 1000 μL of extraction solution/100 mg of tissue. After this process, the suspension obtained was centrifuged at 500*g* for 10 min at 4 °C, and the supernatant was collected, aliquoted, and stored at −80 °C. Cytokine levels (IL-6, IL-10, TNF-α, and IFN-γ) were quantified using OptEIA kits, following the manufacturer’s recommendations (BD Biosciences). Absorbance values were obtained by spectrophotometry at 450 nm, and the concentration of cytokines present in each sample was determined from the linear regression equation obtained from the absorbance values for the standard curve of each cytokine.

### Quantification of Lipid Bodies

For lipid body enumeration, cytospins of bronchoalveolar lavage were prepared using a Cytospin centrifuge (Cytospin, ThermoFisher Scientific), as described previously. The slides were fixed in 3.7% formalin solution for 10 min at room temperature. The cells were then incubated with BODIPY493/503 solution (4,4-difluoro-1,3,5,7,8-pentamethyl-4-bora-3a,4a-diaza-s-indacene) (Molecular Probes, Eugene, OR, USA) at a dilution of 1/10,000 from a stock solution at 1 μg/mL for 1 h at room temperature. The slides were then washed with 1× PBS and mounted with a VECTASHIELD1 mounting medium (Vector Laboratories, Burlingame, CA, USA). The slides were analyzed using a BX-51 fluorescence microscope coupled to an XC-50 digital camera, using a 100x objective lens (Olympus, Tokyo, Japan). Lipid bodies were counted in 50 consecutive cells per slide.

### Analysis of Intestinal Permeability and Macroscopic Alterations

To assess the occurrence of pathological intestinal changes, on the 11th day of the year, mice were fasted for 4 h, then gavaged with 200 μL of FITC-dextran (0.6 mg/g body weight) (fluorescein isothiocyanate–dextran; 4000 kDa; Sigma-Aldrich). After another 4 h in a light-protected environment, the animals were euthanized after anesthesia (100 mg/kg ketamine and 10 mg/kg xylazine), and blood was collected by cardiac puncture, followed by cardiac perfusion and collection of the intestine. To determine intestinal permeability, blood was centrifuged at 3000*g* for 10 min at 4 °C to obtain plasma. Plasma samples were read for fluorescence measurements in triplicate using a plate fluorimeter (Varioskan; ThermoFisher Scientific; 488 nm excitation/530 nm emission), and concentrations of FITC-dextran in plasma were calculated from a standard curve.^28^ Intestinal permeability was also analyzed using the Evans blue staining technique, as previously described.

To determine the macroscopic changes induced by infection, the intestines of animals in each group were measured and photographed.

### Statistical Analysis

Statistical analyses were performed using GraphPad Prism (GraphPad software, version 8.4.3). Survival data were plotted using the Kaplan–Meier method, and statistical differences were evaluated using the log rank test. After assessing data distribution using the Shapiro–Wilk test and homogeneity of variances using the Brown–Forsythe test, data were analyzed using one-way ANOVA followed by Tukey’s multiple comparisons test (for parametric data) or the Kruskal–Wallis test followed by Dunn’s multiple comparisons test (for nonparametric data). If the distribution was parametric, the *Student* *t* test was used to compare the means between two groups. Values of *p* < 0.05 were considered statistically significant.

## Results

### Dietary Supplementation with DHA-Rich Fish Oil Does Not Alter the Clinical Outcome of Infection and Is Associated with High Rates of Circulating Parasites

To determine the clinical profile and survival, the animals received dietary supplementation with DHA-rich fish oil (3 g of DHA/kg) for 15 days and were then infected with PbN. Supplementation was maintained until the animals died spontaneously. Both supplemented/infected animals (DHA/PbN) and those infected only (PbN) showed progressive clinical deterioration throughout the follow-up (Figure 1A), with dehydration, hair erection, urinary incontinence, body curvature, and loss of muscle tone being the main signs (Figure S2). As can be seen, dehydration and piloerection were the first signs observed, reaching maximum scores around the 14th and 9th d.p.i, respectively, in both groups. On the other hand, the maximum degree of incontinence was only observed in the last days of infection. In general, clinical deterioration was accompanied by an increase in systemic parasitemia, which was particularly increased in the DHA/PbN group compared to the PbN group only on days 9, 10, and 11 p.i. On day 22 p.i., the circulating parasitemia rate in both groups peaked at over 50%, characterizing the typical hyperparasitemia (Figure 1B); at this point in the follow-up, 100% of the animals succumbed to death (Figure 1C).

**Figure 1:**
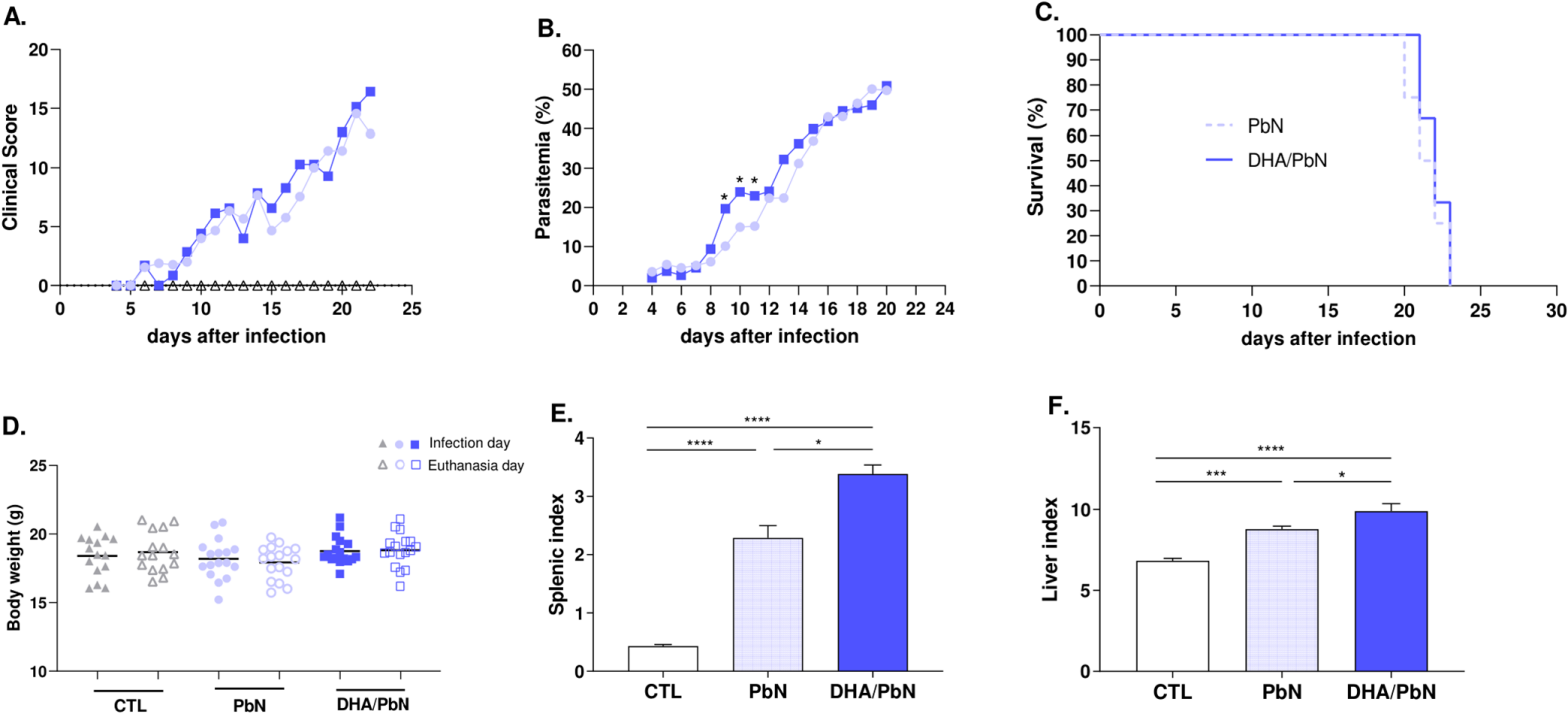
Assessment of the clinical conditions of mice, systemic parasitemia, survival, body weight, spleen, and liver indices of mice supplemented with DHA-rich fish oil and infected with PbN. (A) Clinical assessment of animals throughout infection; (B) blood parasitemia from 4th to 22nd d.p.i.; (C) survival curve of supplemented and infected animals and infected animals only; (D) body weight of animals on the day of infection and on the day of euthanasia (11th d.p.i.); (E) splenic index (11th d.p.i.); (F) hepatic index (11th d.p.i.). Results are shown as frequency or mean ± SEM of two independent experiments. (*) indicates statistical difference: *p < 0.05; ***p < 0.001; ****p < 0.0001. CTL: unsupplemented and uninfected mice (triangle); PbN: mice infected with *P. berghei* NK65 (circle); DHA/PbN: mice supplemented with 3 g/kg of DHA and infected with PbN (square).

Since, from the 12th dpi, the circulating parasite rates were similar between the PbN and DHA/PbN groups, the 11th dpi was chosen for euthanasia and organ collection for additional experiments. As illustrated in Figure 1D, oil supplementation did not alter the weight of the animals compared to those that did not receive supplementation. On the other hand, animals belonging to the DHA/PbN group had significantly increased spleen and liver indices compared to control and PbN animals (Figure 1E,F). TGO and TGP levels were also significantly elevated in infected animals, regardless of whether they were supplemented (Figure S3).

### Dietary Supplementation with DHA-Rich Fish Oil Does Not Mitigate Hematological and Pulmonary Injury Caused by PbN Infection in C57BL/6 Mice

Considering previous studies conducted with the same strain of mice infected with PbN,^26,27^ as well as the difference in circulating parasite levels between days 9 and 11 p.i. observed between infected experimental groups, for all subsequent analyses, the animals were euthanized on the 11th d.p.i.. On that day, the main clinical signs observed in the PbN and DHA/PbN groups were dehydration (mean score = 4) and piloerection (mean score = 1). In these groups, there was a significant increase in the number of circulating leukocytes compared to that observed in the control animals. On the other hand, erythrocyte and platelet counts, as well as serum hemoglobin (g/dL) and hematocrit (%) levels, were significantly reduced in both the PbN and DHA/PbN groups compared to the control (Table 1).

**Table 1:** Hematological Analyses of Mice Supplemented with DHA-Rich Fish Oil and Infected with PbN^a^

		groups		
parameters	CTL	PbN	DHA/PbN	*P* value
total leukocytes (10^3^/μL)	4.72 (±1.53)^a^	6.54 (±2.23)^b^	7.80 (±2.78)^b^	0.0046
erythrocytes (10^6^/μL)	8.41 (±0.68)^a^	5.61 (±0.89)^b^	4.93 (±1.29)^b^	<0.0001
platelets (10^3^/μL)	502.36 (±117.25)^a^	95.7 (±18.56)^b^	92.0 (±23.77)^b^	<0.0001
hematocrit (%)	42.95 (±3.65)^a^	29.81 (±3.89)^b^	29.70 (±5.10)^b^	0.0002
hemoglobin (g/dL)	12.80 (±1.0)^a^	7.99 (±1.23)^b^	6.90 (±1.91)^b^	<0.0001

a Results, representative of two independent experiments (*n* = 5–6 mice/group), are shown as mean (±SEM). CTL: unsupplemented and uninfected mice; PbN: mice infected with *P. berghei* NK65; DHA/PbN: mice supplemented with 3 g DHA/kg and infected with PbN. Differences between columns are indicated by letters.

Next, we investigated the occurrence of pulmonary injury, such as vascular permeability and edema (in nonperfused animal lungs), induced by PbN. As observed, animals from PbN and DHA/PbN groups showed a significant increase in alveolar vascular permeability (Figure 2A,B) compared to control animals, corroborating the presence of pulmonary edema (Figure 2C) in both groups. Similarly, the concentration of proteins in BAL was significantly increased in the infected groups, PbN and DHA/PbN, when compared to that in control animals (Figure 2D). In both groups, histopathological analysis showed mild to moderate inflammation with intravascular leukocyte margination (arrow) and mild thickening of the interalveolar septa (arrowhead) (Figure 2E).

**Figure 2:**
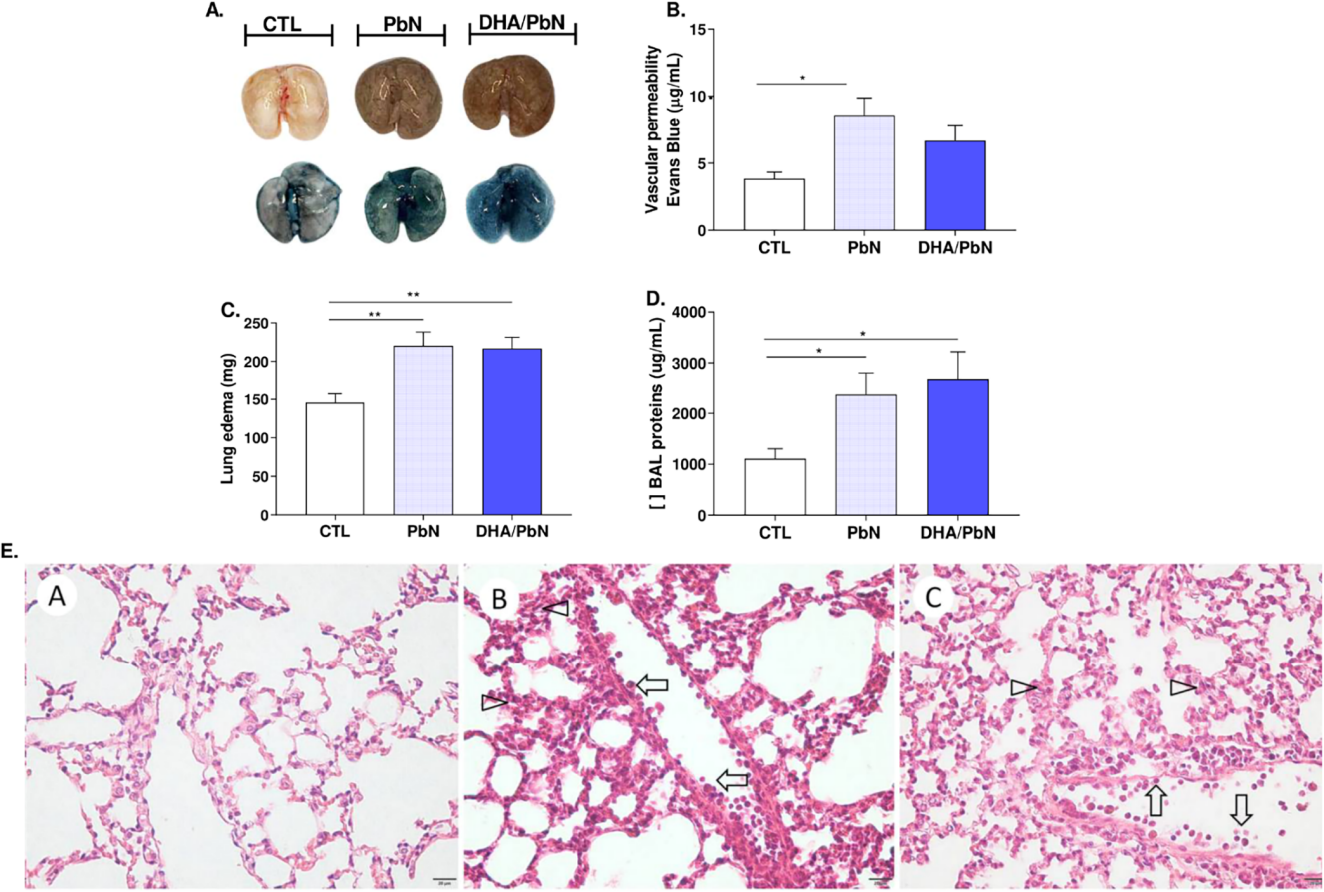
Analysis of vascular permeability, pulmonary edema, protein concentration, and lung histology in mice supplemented with DHA-rich fish oil and infected with PbN. (A) Lungs stained with Evans blue dye; (B) concentration of extravasated Evans blue dye (μg/mL); (C) lung weight (mg); (D) total protein concentration in BAL supernatant (μg/mL); (E) photomicrographs of lung tissue: (A) CTL: uninfected control group, (B) PbN: infected animals only, (C) DHA/PbN: supplemented and infected animals. Mild to moderate inflammation, intravascular leukocyte margination (arrow), thickening of interalveolar septa (arrowhead). Calibration bar of 20 μm. The results are representative of three independent experiments (*n* = 5–6 mice/group) and are shown as mean ± SEM. (*) indicates statistical difference: *p < 0.05; **p < 0.01.

### Dietary Supplementation with DHA-Rich Fish Oil Does Not Prevent Inflammatory Cell Infiltration and Oxidative Stress in the Lungs from C57BL/6 Mice Infected with PbN

The pulmonary pathology induced by experimental infection with PbN was also investigated by collecting and analyzing BAL obtained on day 11 p.i. (Figure 3). Initially, the total number of cells was determined, which was significantly higher in infected animals compared to the control group regardless of fish oil supplementation (Figure 3A). Among the cell types infiltrating the lungs, macrophages were predominant (Figure 3B). Finally, the influence of oxidative stress on lung damage can be suggested based on the levels of hydrogen peroxide (H_2_O_2_) produced by cells obtained from BAL (Figure 3C). In this case, there was no difference between the levels of H_2_O_2_ produced by cells from infected animals.

**Figure 3:**
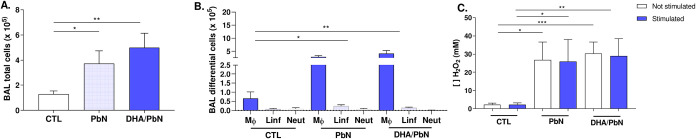
Evaluation of inflammatory infiltrate and H_2_O_2_release in bronchoalveolar lavage fluid from mice supplemented with DHA-rich fish oil and infected with PbN. (A) Total cell concentration in BAL; (B) differential count of cell populations in BAL; (C) concentration of H_2_O_2_ produced by inflammatory cells present in BAL (mM). The graphs A and B show the mean number of cells (×10^5^). The results are representative of two independent experiments (*n* = 5–6 mice/group) and are shown as mean ± SEM. (*) indicates statistical difference: *p < 0.05; **p < 0.01; ***p < 0.001. CTL: unsupplemented and uninfected mice; PbN: mice infected with *P. berghei* NK65; DHA/PbN: mice supplemented with 3 g/kg of DHA and infected with PbN.

### Dietary Supplementation with DHA-Rich Fish Oil Does Not Alter the Phenotypic Profile of Lung Cells Observed during PbN Infection in C57BL/6 Mice

Next, the phenotypic profile of lung cells in infected animals, supplemented or not with fish oil containing 3 g of DHA/kg of body weight, was investigated. Corroborating the anatomopathological findings, it was observed that the total number of cells for all subtypes analyzed (leukocytes, myeloid cells, inflammatory monocytes, T lymphocytes, CD4 T lymphocytes, and CD8 T lymphocytes) was significantly increased in infected animals, PbN and DHA/PbN, compared to the controls. Interestingly, a trend toward a greater increase in the number of total leukocytes, myeloid cells, and inflammatory monocytes was observed for animals in the DHA/PbN group compared to the PbN group (Figure 4A–F).

**Figure 4:**
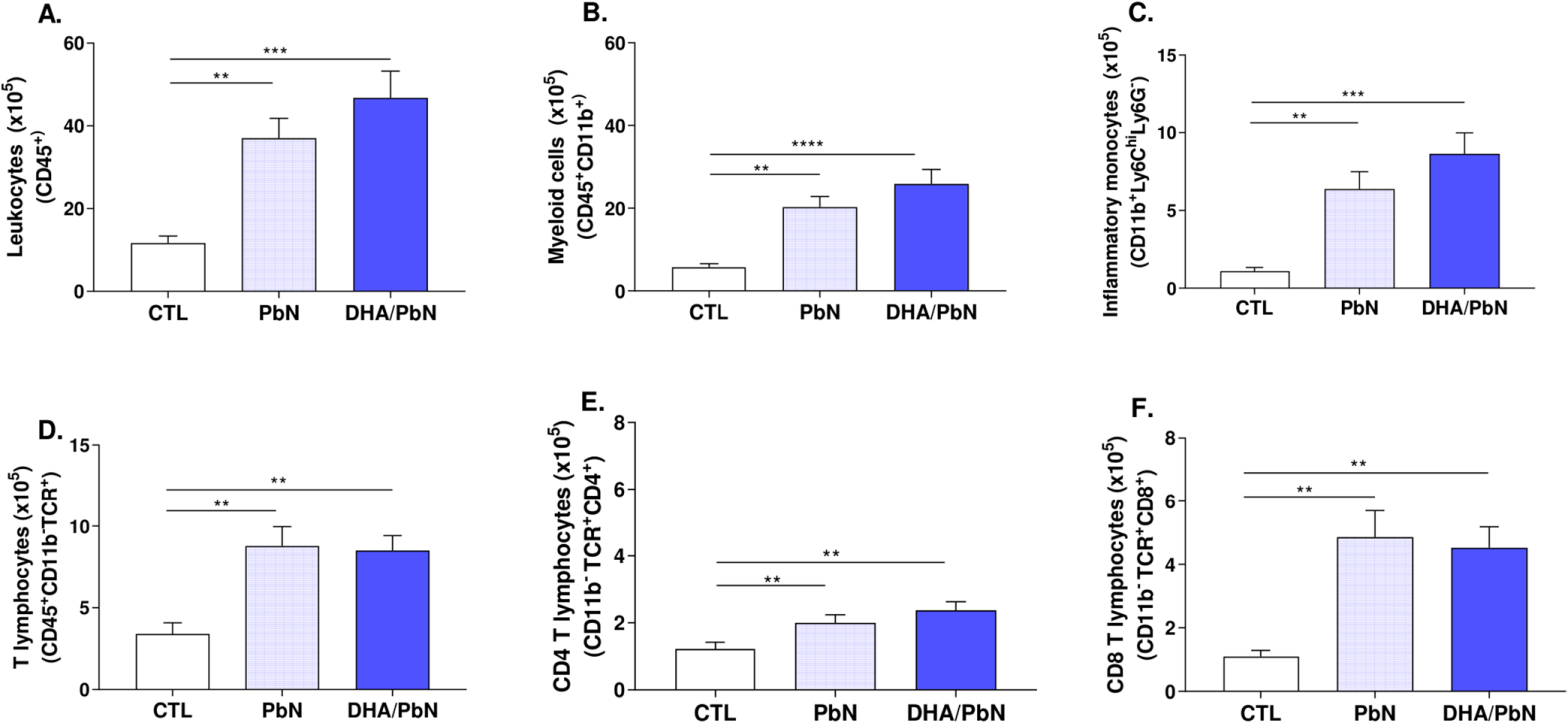
Evaluation of the phenotypic profile of cell populations present in the lung tissue of mice supplemented with DHA-rich fish oil and infected with PbN. (A) Total leukocytes; (B) myeloid cells; (C) inflammatory monocytes; (D) T lymphocytes; (E) CD4 T lymphocytes; (F) CD8 T lymphocytes. The graphs show the mean number of cells expressing the specific receptor(s) (×10^5^). Results are representative of 5–6 mice per group and are shown as mean ± SEM. (*) indicates statistical difference: **p < 0.01; ***p < 0.001; ****p < 0.0001. CTL: unsupplemented and uninfected mice; PbN: mice infected with *P. berghei* NK65; DHA/PbN: mice supplemented with 3 g/kg DHA and infected with PbN.

### Dietary Supplementation with DHA-Rich Fish Oil Does Not Alter the Pattern of Pulmonary Inflammatory Response Induced by Experimental Infection with PbN

The dosage of pro-inflammatory and anti-inflammatory cytokines in lung tissue was measured using an enzyme-linked immunosorbent assay (ELISA). It was observed that pro-inflammatory cytokine levels were significantly elevated in infected animals, PbN, and DHA/PbN, when compared to the control group (Figure 5A–C). The same was observed for the anti-inflammatory cytokine IL-10, where concentrations were significantly increased in the infected groups regardless of supplementation (Figure 5D).

**Figure 5:**
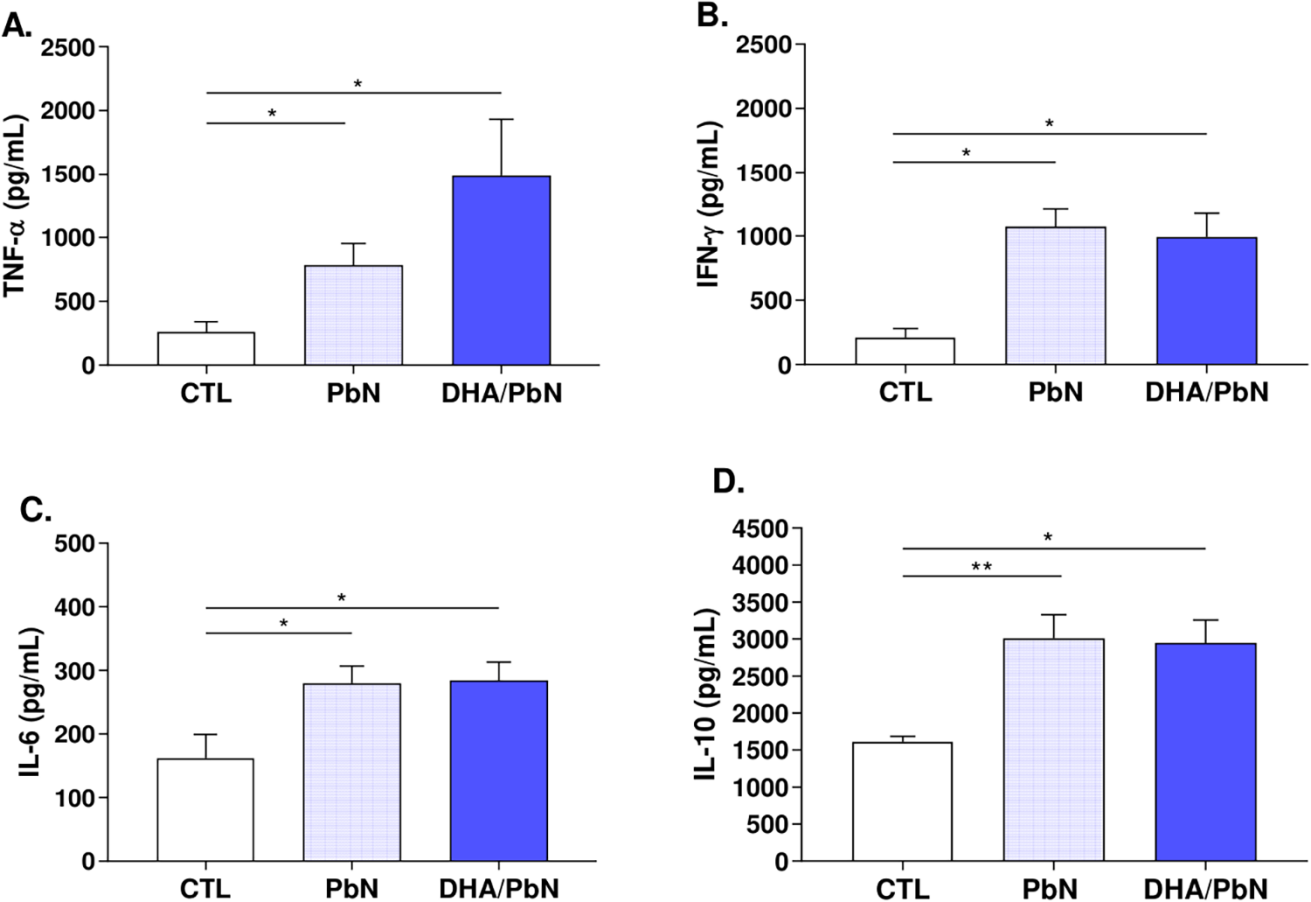
Dosage of pro-inflammatory and anti-inflammatory cytokines in lung tissue homogenate from mice supplemented with DHA-rich fish oil and infected with PbN. (A) TNF-α levels (pg/mL); (B) IFN-γ (pg/mL); (C) IL-6 (pg/mL); (D) IL-10 (pg/mL). The results are representative of two independent experiments (*n* = 5–6 mice/group) and are shown as mean ± SEM. (*) indicates statistical difference: *p < 0.05; **p < 0.01. CTL: unsupplemented and uninfected mice; PbN: mice infected with *P. berghei* NK65; DHA/PbN: mice supplemented with 3 g/kg DHA and infected with PbN.

### Dietary Supplementation with DHA-Rich Fish Oil Was Associated with Reduced Formation of Lipid Bodies in Bronchoalveolar Lavage Cells during PbN Infection in C57BL/6 Mice

The analysis of lipid body formation was performed after staining the cytospin with fluorescent probe BODIPY493/503. The counts showed that there was a small but significant increase in the number of these organelles in the PbN-infected group (Figure 6B) compared to the uninfected control CTL (Figure 6A). Treatment with DHA was associated with a decrease in the level of formation of these organelles (Figure 6D). The frequency distribution of lipid droplets in Figure 6E shows that in all groups most cells have few lipid droplets. However, the infected PbN group has some cells with a high number of these organelles, which contributed to the increase in the group average (Figure 6D).

**Figure 6:**
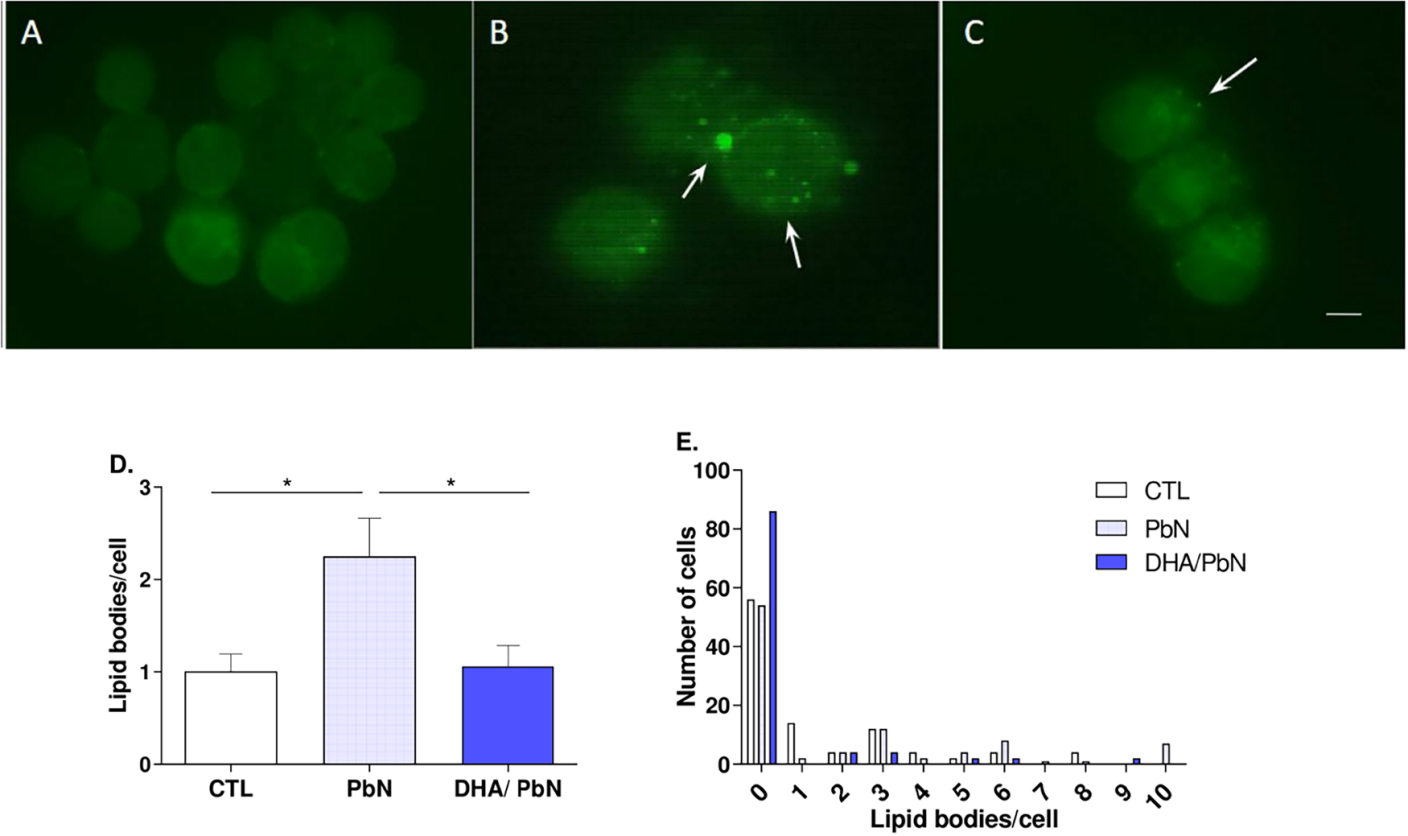
Analysis of lipid body formation in bronchoalveolar lavage cells from mice supplemented with DHA-rich fish oil and infected with PbN. (A–C) Photomicrographs of bronchoalveolar lavage smears from (A) CTL: uninfected control group, (B) PbN: infected animals only, (C) DHA/PbN: supplemented and infected animals. Lipid bodies stained with BODIPY are observed in the PbN and DHA/PbN groups (white arrows). Bar = 10 μm. (D) Quantification of lipid bodies in cells from the CTL, PbN, and DHA/PbN groups; (E) frequency distribution of lipid bodies per cell. One hundred cells per group were analyzed. Results are expressed as mean ± SEM from two independent experiments, where lipid bodies were counted from 50 cells per animal (*n* = 5–6 mice/group). (*) indicates statistical difference: *p < 0.05.

### Dietary Supplementation with DHA-Rich Fish Oil Prevents Morphometric Changes but Does Not Interfere with the Increase in Intestinal Permeability Induced by PbN Infection in C57BL/6 Mice

To investigate the possible impact of experimental PbN infection on the intestinal tract (Figure 7), animals belonging to each of the experimental groups were euthanized on the 11th dpi. Macroscopically, a significant reduction in the total length of the intestine (Figure 7A), small intestine (Figure 7B), colon (Figure 7C), and cecum (Figure 7D) was observed in infected animals when compared to those of supplemented and control groups. Although the difference was not statistically significant, the data from two different assays indicated a trend toward reduced intestinal permeability in the DHA/PbN group compared with that in the PbN group (Figure 7E,F).

**Figure 7:**
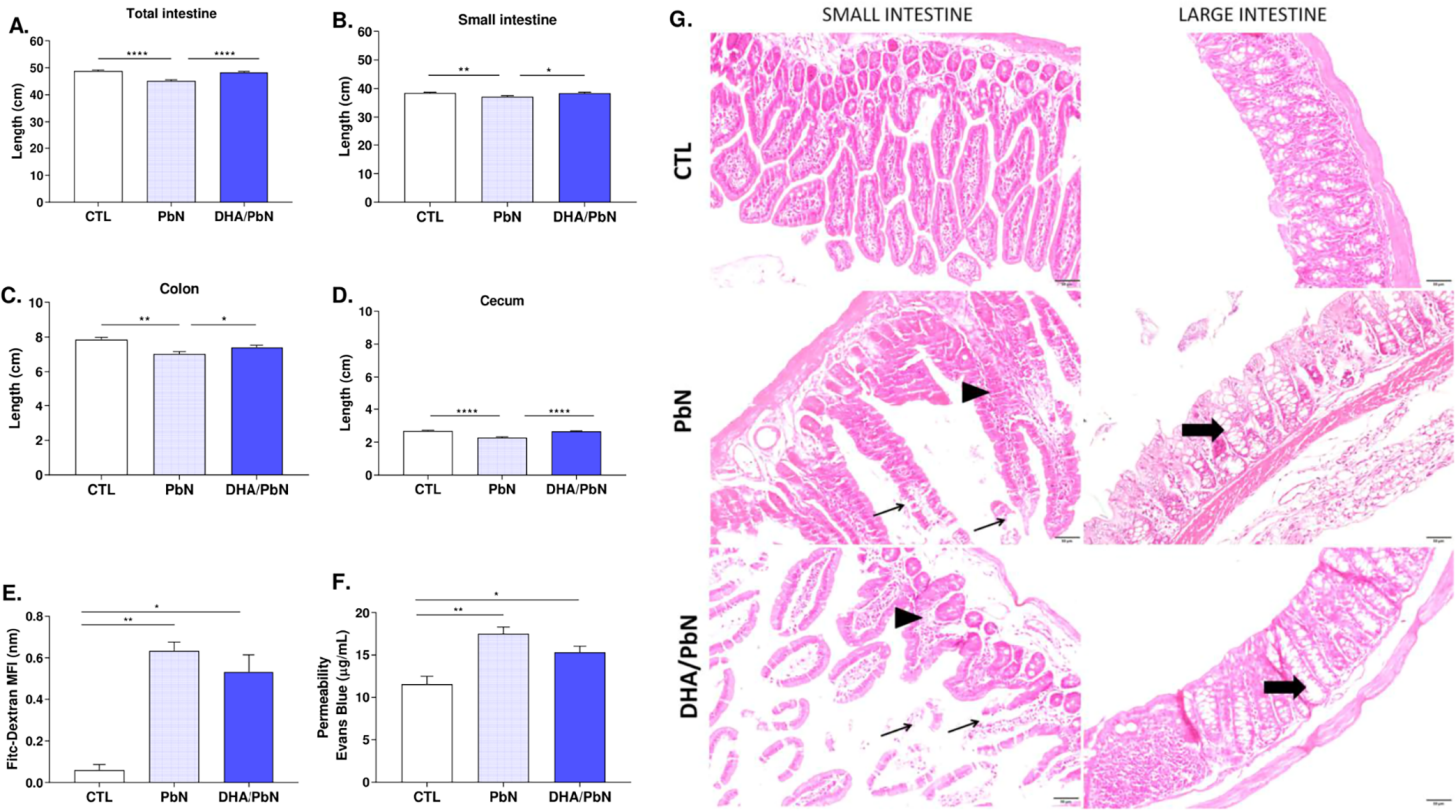
Assessment of intestinal alterations in mice supplemented with DHA-rich fish oil and infected with PbN. (A) Total intestine length (cm); (B) small intestine length (cm); (C) colon length (cm); (D) cecum length (cm); (E) average fluorescence intensity of plasma FITC-Dextran; (F) evans blue concentration in the tissue (μg/mL); (G) photomicrographs of intestine tissue, showing villous blunting or fusion (arrowhead), loss of enterocytes (thin arrow) and crypt rarefaction (thick arrow). Calibration bar of 50 μm. The results are representative of two independent experiments (*n* = 5–6 mice/group) and are shown as mean ± SEM. (*) indicates statistical difference: *p < 0.05; **p < 0.01; ****p < 0.0001. CTL: unsupplemented and uninfected mice; PbN: mice infected with *P. berghei* NK65; DHA/PbN: mice supplemented with 3 g/kg DHA and infected with PbN.

Microscopically (Figure 7G), the infected group presented tissue lesions that ranged from mild to moderate. Small intestine lesions included villous blunting and fusion with vacuolization and loss of enterocytes. The main large intestine lesion was crypt rarefaction. The DHA/PbN group showed improvement in tissue integrity, and lesions were milder compared to those in the infected unsupplemented group (PbN).

## Discussion

Serious complications arising from malaria infection, such as cerebral malaria, severe anemia, and acute respiratory distress syndrome (ARDS), are determinants of the disease’s prognosis. ARDS, which mainly affects adults, results from an intense inflammatory response in the lung parenchyma, culminating in increased alveolar vascular permeability, edema, and consequent impairment of gas exchange, with the possibility of progression to death (20% of cases).^29^ Recognition of the relevance of this complication is essential to guide research that seeks to understand its pathophysiological mechanisms and proposes more effective therapeutic alternatives. We recently demonstrated that C57BL/6 mice supplemented with fish oil containing high concentrations of DHA were protected against severe pulmonary injury caused by experimental malaria infection with *Pb*. ANKA.^24^ In this study, however, we demonstrated that the results obtained in our previous study cannot be extrapolated to other strains of parasites capable of inducing lung lesions.

Initially, we showed that PbN and DHA/PbN groups had a mortality rate of 100% between days 20 and 22 of infection, corroborated by progressive clinical deterioration throughout the follow-up and by an increase in systemic parasitemia, which, surprisingly, was higher in animals supplemented between days 9 and 11 p.i. These findings contrast with previous studies that showed that supplementation with fish oil containing or not containing antioxidants such as vitamin E was able to suppress parasitemia in vitro assay using *P. falciparum* ^22,23^ and, in vivo experimental infections with *P. berghei* ^30^ and *P. yoelii*.^31,32^ However, it is important to emphasize that experiments previously conducted by other groups differ in experimental strategy, such as dose, route, and time of administration, which makes the results not strictly comparable. In any case, using strictly the same experimental protocol, our group recently demonstrated that supplementation with DHA-rich ω-3 was able to control circulating parasitemia in C57BL/6 mice infected with PbA^24^ and that supplementation with high doses of DHA reduced the risk of cerebral malaria by 60% to 80%,^25^ as well as, protecting the animals against severe lung injury.^24^ No significant changes in body weight were observed in animals receiving supplementation, which is consistent with previous studies that also found no relationship between dietary fish oil supplementation and weight gain in either rodents^33,34^ or humans.^35,36^

In addition, other common observations during malaria infections include hematological changes and enlargement of organs such as the spleen and liver,^37,38^ which are usually associated with the intense inflammatory response generated by the body in response to the presence and/or persistence of blood parasitemia. In this study, animals in the PbN and DHA/PbN groups showed significant hepatosplenomegaly. Regarding hematological changes, we found that infection with PbN led to a significant increase in the number of leukocytes in the peripheral blood, which was not mitigated by supplementation. The marked reduction in the number of erythrocytes and, consequently, hematocrit and hemoglobin concentration in the blood of infected animals, supplemented or not, compared to uninfected animals, corroborates previous studies suggesting intense destruction of red blood cells, parasitized or not, in malaria infection^37^ and that this destruction is associated with changes in the membrane of these cells and the expression of parasite proteins that induce apoptosis. Additionally, the adsorption of antigens and metabolites from the parasites in healthy red blood cells contributes significantly to the destruction of these cells by the spleen.^38^ Finally, the production of hemozoin by parasites, as well as the immune response characterized by intense production of pro-inflammatory cytokines, also contribute to bone marrow dysfunction and, consequently, anemia.^39^

In the same context, we observed a significant reduction in the number of circulating platelets in the infected groups, independent of supplementation. It is already known that thrombocytopenia is a common hematological manifestation in malaria, especially when caused by *P. vivax*.^40^ Given this, our data corroborate the results previously described by De Azevedo-Quintanilha and colleagues, in which animals infected with PbN presented thrombocytopenia on the ninth day of infection, possibly due to the formation of cell aggregates, such as platelet-monocytes and platelet-neutrophils,^41^ with no indication that supplementation alters these outcomes. Another hypothesis for thrombocytopenia is platelet sequestration in the spleen, as a result of specific binding of IgG to platelet-bound malaria antigens.^42,43^

Next, in line with the main goal of this study, we investigated the impact of suplemmentation with ω-3 on the lungs from animals infected with PbN. Pulmonary edema, characterized by increased vascular permeability and accumulation of alveolar fluid, is known to be the most common manifestation of malaria-associated lung injury.^26,41^ In our study, increased vascular permeability and consequent pulmonary edema were observed, as well as a higher concentration of proteins in the bronchoalveolar lavage supernatant in both infected groups, suggesting that DHA-rich oil fish supplementation was not able to act against the mechanisms that trigger these processes. Our model, however, appears to be appropriate, as it corroborates previous studies that showed that mice infected with PbN have increased vascular permeability and pulmonary edema from day 8 p.i.,^27,29^ in addition to an influx of inflammatory cells into the tissue, contributing to pulmonary lesions.^27^ In inflammatory processes, monocytes are one of the first cell populations to migrate to the tissue, playing a prominent role in the pathology of malaria, since they differentiate into macrophages and act in the processes of phagocytosis, antigen presentation, and cytokine production.^44^ It is known that activated macrophages have as their main microbicidal mechanism the production of reactive oxygen species (ROS) and reactive nitrogen species (RNS), such as hydrogen peroxide (H_2_O_2_) and nitric oxide (NO), respectively, and are important for eliminating the parasite through an oxidative burst; however, they are responsible for causing intense oxidative damage to cell membranes. Our study shows that there is significant production of H_2_O_2_ by cells in the lungs of infected animals, regardless of supplementation, suggesting that inflammatory cells are overactivated in these animals and that DHA-rich fish oil is unable to act on the pathways that control the inflammatory response of animals infected with PbN.

To better characterize the inflammatory response, the phenotypic profile of leukocytes in lung and spleen tissues was analyzed. Our data showed that there was a significant infiltration of these cells in both organs of the infected groups, especially inflammatory monocytes and T lymphocytes (CD8+ and CD4+). Although there was no significant difference, the supplemented group showed a tendency toward greater cell recruitment in the myeloid compartment in the lung compared to the PbN group (Figure 4A–C). In contrast, in the spleen, there was a tendency toward a reduction in T lymphocytes, both CD4+ and CD8+ (Figure S4C–E), in animals that received supplementation compared to those only infected. Previous studies have shown that in PbN murine malaria, there is a 20-fold increase in the number of CD8+ T lymphocytes infiltrating the lungs 10 days after infection and that these cells appear to contribute significantly to the development of pulmonary pathology, since their deletion with anti-CD8 antibodies leads to a reduction in alveolar edema and cellular infiltration.^29^ These results are surprising to us, since in a previous infection model with PbA conducted by our group supplementation with DHA-rich fish oil significantly reduced the number of inflammatory monocytes and CD8^+^ T lymphocytes in both the lungs and brains of infected animals.^24,25^

It is also known that the balance between pro- and anti-inflammatory cytokines is essential for controlling infection and returning to homeostasis.^45^ In this study, we observed an increase in the production of the pro-inflammatory cytokines TNF-α, IFN-γ, and IL-6 in the lungs of infected animals and that DHA-rich fish oil supplementation was unable to alter this profile. These data are consistent with previous studies that have shown that the main source of these cytokines is macrophages and activated lymphocytes and that the severity of the disease is associated with the production of pro-inflammatory cytokines in both murine^46^ and human^47^ models. Really, a recent study showed that PbN-induced malaria was characterized by increased levels of TNF-α, IFN-γ, and IL-1β from the seventh d.p.i.^27^ In the same way, PbN and DHA/PbN animals had elevated levels of IL-10 when compared with the control group. In general, increased levels of this cytokine are associated with anti-inflammatory effects.^48–50^ However, Taylor and colleagues showed that elevated levels of IL-10 in adult patients with falciparum malaria were associated with a worse clinical outcome, which could culminate in death in severe cases without cerebral involvement.^51^ Furthermore, increased levels of IL-10 at the onset of ARDS development not associated with malaria are related to a worse prognosis of the disease.^52^ Similarly, an excessive increase in IL-10 production was observed in a murine infection model induced by *P. berghei* K173, but not by PbA, while blocking this cytokine prevented the development of MA-ARDS. Mukherjee and colleagues also found that infection and exacerbated IL-10 production were associated with an uncontrolled antimicrobial immune response, resulting in pulmonary dysbiosis, which is directly related to the development of the clinical condition.^53^ Although we did not evaluate parameters associated with the microbiota, it is possible to hypothesize that both PbN and Pb K173 lead to the development of ARDS through alteration of the pulmonary microbiota. Therefore, further studies are needed to understand the mechanisms associated with ARDS immunopathology in the presence of different strains/species of *Plasmodium*.

Next, we also evaluated the formation of lipid bodies in bronchoalveolar lavage cells. Lipid bodies are sites of eicosanoid synthesis, such as prostaglandins and leukotrienes, especially under the conditions of inflammation and infection.^54^ At high concentrations, prostaglandin E_2_ (PGE_2_) is a potent inhibitor of Th1-type responses and of TNF and NO production and, therefore, dampens the inflammatory response.^55^ Here, we observed that *P. berghei* NK65 infection induced a modest but statistically significant formation of lipid bodies, while supplementation with DHA-rich fish oil was associated with inhibition of this formation. We can hypothesize that this inhibition of lipid body formation may have contributed to the reduction in eicosanoid synthesis, such as PGE_2_, which coincided with the slight increase in TNF-α synthesis. Indeed, it has been suggested that induction of COX-2 expression and prostaglandin synthesis may play a protective role against the worsening of malarial infection.^56^ On the other hand, leukotrienes are pro-inflammatory molecules that originate from another metabolic pathway of arachidonic acid. The production of these arachidonic acid–derived molecules with opposing functions depends on the cellular pathway that is stimulated: the cyclooxygenase pathway produces prostaglandins, whereas the lipoxygenase pathway produces leukotrienes.^56^ A previous study showed that 5-lipoxygenase–deficient mice infected with *P. berghei* ANKA exhibited increased cerebral inflammation and, consequently, early death,^57^ suggesting that this pathway also plays a role in the progression of *P. berghei* ANKA infection. Our data suggest that the reduction in the number of lipid bodies associated with DHA-rich fish oil supplementation and a consequent decrease in eicosanoid production derived from these organelles may be related to the increased parasitemia observed in this group of animals. Nevertheless, further studies are needed to confirm this hypothesis.

Finally, we also demonstrated that the NK65 strain of *P. berghei*, similar to the ANKA strain,^28^ is capable of causing intestinal changes (by both macroscopic and histopathological analyses), altering intestinal permeability. Although no statistically significant difference has been observed in intestinal permeability assessed by two distinct techniques (FITC–dextran quantification and Evans blue assay), both methods indicated a trend toward reduced permeability in animals supplemented with DHA-rich fish oil compared to the PbN group. This observation is consistent with the finding that supplemented animals did not exhibit intestinal shortening and displayed milder histopathological alterations compared with animals in the PbA group. Nevertheless, further studies are required to investigate the relationship between omega-3 intake, its effects on the intestinal environment, and malaria infection, especially to validate our findings and elucidate the mechanisms involved. Previous studies have shown that increased intestinal permeability favors dysbiosis and the translocation of bacteria into the lamina propria, disrupting tolerance to dietary and microbial antigens and ultimately leading to immune activation and inflammation of the intestinal mucosa.^28,58^ The pathways through which ω-3 components may act to mediate such protection need to be investigated. Robertson and colleagues have shown that dietary supplementation with ω-3 promotes the growth of bacteria that produce short-chain fatty acids, such as butyrate and acetate.^59^ Butyrate, for example, is seen to have an anti-inflammatory effect, since it acts by inhibiting the activation of NF-κB,^60^ among other mechanisms. Acetate, on the other hand, in an intestinal model, appears to act by increasing the expression of barrier genes (such as claudin 4), in addition to modulating the expression of defensins, reducing oxidative stress in the intestine.^61^ However, in the malaria model, these mechanisms still remain to be clarified.

In summary, our study demonstrates that supplementation with DHA-rich fish oil was not able to mitigate pulmonary damage induced by malarial infection in the PbN model, although it was associated with the prevention of intestinal shortening. Despite the recognized relevance of the gut–lung axis in the pathophysiology of respiratory diseases, the results presented here do not support the inference of a direct mechanistic relationship between the alterations observed in these organs. An additional consideration is that, although the DHA dose employed in this study is higher than those typically used in clinical settings,^62–64^ it was selected based on our prior dose–response experiments demonstrating biological efficacy. Nevertheless, the translational relevance of this high-dose regimen should be interpreted with caution, as interspecies differences in pharmacokinetics, metabolism, and tolerability may influence both the magnitude and direction of the observed effects. Finally, our findings highlight the importance of considering the variability in pathogenic profiles among *Plasmodium* strains when evaluating nutritional or immunomodulatory interventions. These observations reinforce that potential adjuvant strategies for malaria management must be carefully validated across different experimental models before extrapolation to clinical settings.

## Supplementary Material



## Data Availability

All data generated or analyzed during this study are included in this article. The data underlying this study are available in the published article and its Supporting Information.

## Notes

Nutritional information for the super concentrated DHA oil supplement (Table S1); gating strategy for phenotyping pulmonary and splenic cells (Figure S1A–B); clinical score of animals supplemented or not with fish oil and infected or not with PbN (Figure S2A–E); plasma levels of TGO and TGP (Figure S2A–B); and phenotypic profile of splenic cells (Figure 4A–E) (PDF)

## Notes

This research was funded by the *Fundação de Amparo à Pesquisa do Estado de Minas Gerais* (FAPEMIG—*Rede Mineira* #00198/23 and APQ-1365-22), *Conselho Nacional de Desenvolvimento Científico e Tecnológico* (CNPq 310272/2022-0). L.P.M.C.P. received UFJF fellowship; J.H.S.R., F.M.P., and C.B.M. received CAPES fellowships; M.T.G. received FAPEMIG fellowships. The authors thank the support offered by the Universidade Federal Juiz de Fora and Integrated Research Laboratory of *Programa de Pós-Graduação em Ciências Biológicas* of the Universidade Federal de Juiz de Fora. The graphical abstract was created using BioRender.

## References

[1] World Health Organization . World Malaria Report 2024: Addressing Inequity in the Global Malaria Response; World Health Organization, 2024.

[2] Schofield L., Grau G. E. (2005). Immunological processes
in malaria pathogenesis. Nat. Rev. Immunol..

[3] Nguee S. Y.
T., Júnior J.W.B.D., Epiphanio S., Rénia L., Claser C. (2022). Experimental models
to study the
pathogenesis of malaria-associated acute respiratory distress syndrome. Front. Cell. Infect. Microbiol..

[4] Taylor W. R., White N. J. (2002). Malaria and the
lung. Clin. Chest
Med..

[5] Taylor W. R., Hanson J., Turner G. D., White N. J., Dondorp A. M. (2012). Respiratory
manifestations of malaria. Chest.

[6] Mohan A., Sharma S. K., Bollineni S. (2008). Acute lung injury and acute respiratory
distress syndrome in malaria. J. Vector Borne
Dis..

[7] Deroost K., Tyberghein A., Lays N., Noppen S. (2013). Hemozoin
induces lung inflammation and correlates with malaria-associated acute
respiratory distress syndrome. Am. J. Respir.
Cell Mol. Biol..

[8] Claser C., Malleret B., Peng K., Gun S. Y. (2014). Rodent
Plasmodium-infected red blood cells: Imaging their fates and interactions
within their hosts. Parasitol. Int..

[9] Claser C., Nguee S. Y. T., Balachander A., Wu Howland S. (2019). Lung endothelial cell antigen cross-presentation
to CD8+ T cells
drives malaria-associated lung injury. Nat.
Commun..

[10] Possemiers H., Pham T. T., Coens M., Pollenus E., Knoops S. (2021). Skeleton binding protein-1-mediated parasite sequestration
inhibits
spontaneous resolution of malaria-associated acute respiratory distress
syndrome. PLoS Pathog..

[11] Dolinay T., Kim Y. S., Howrylak J., Hunninghake G. M. (2012). Inflammasome-regulated cytokines are critical
mediators of acute
lung injury. Am. J. Respir. Crit. Care Med..

[12] Mills C. D., Kincaid K., Alt J. M., Heilman M. J., Hill A. M. (2000). M-1/M-2
Macrophages and the Th1/Th2 Paradigm. J. Immunol..

[13] D’Ambrosio D., Mariani M., Panina-Bordignon P., Sinigaglia F. (2001). Chemokines
and their receptors guiding T lymphocyte recruitment in lung inflammation. Am. J. Respir. Crit. Care Med..

[14] Fuhlbrigge R. C., Alon R., Puri K. D., Lowe J. D., Springer T. A. (1996). Sialylated,
fucosylated ligands for L-selectin expressed on leukocytes mediate
tethering and rolling adhesions in physiologic flow conditions. J. Cell Biol..

[15] González-Amaro R., Sánchez-Madrid F. (1999). Cell adhesion
molecules: selectins
and integrins. Crit. Rev. Immunol..

[16] Lawrence M. B. (1999). Selectin–carbohydrate
interactions in shear flow. Curr. Opin. Chem.
Biol..

[17] Budden K. F., Gellatly S. L., Wood D. L., Cooper M. A. (2017). Emerging
pathogenic links between microbiota and the gut–lung axis. Nat. Rev. Microbiol..

[18] Calder P. C. (2017). Omega-3
fatty acids and inflammatory processes: from molecules to man. Biochem. Soc. Trans..

[19] Vega O. M., Abkenari S., Tong Z., Tedman A., Huerta-Yepez S. (2021). Omega-3 Polyunsaturated
Fatty Acids and Lung Cancer: Nutrition or Pharmacology?. Nutr. Cancer.

[20] Singer P., Shapiro H. (2009). Enteral omega-3 in acute respiratory
distress syndrome. Curr. Opin. Clin. Nutr. Metab.
Care.

[21] Alhusseiny S. M., El-Beshbishi S. N. (2020). Omega polyunsaturated fatty acids
and parasitic infections:
An overview. Acta Trop..

[22] Kumaratilake L. M., Robinson B. S., Ferrante A., Poulos A. (1992). Antimalarial properties
of n-3 and n-6 polyunsaturated fatty acids: in vitro effects on *Plasmodium falciparum* and in vivo effects on *P. berghei*. J. Clin. Invest..

[23] Ommi N. B., Abdullah M., Guruprasad L., Babu P. P. (2022). Docosahexaenoic
acid is potent against the growth of mature stages of *Plasmodium falciparum*; inhibition of hematin polymerization
a possible target. Parasitol. Int..

[24] David-Vieira C., Carpinter B. A., Bezerra-Bellei J. C., Machado L. F. (2024). Lung
Damage Induced by Plasmodium berghei ANKA in Murine Model of Malarial
Infection is Mitigated by Dietary Supplementation with DHA-Rich Omega-3. ACS Infect. Dis..

[25] Carpinter B. A., Renhe D. C., Bezzera-Bellei J. C., David-Vieira C. (2024). DHA-rich fish oil plays a protective role against
experimental cerebral
malaria by controlling inflammatory and mechanical events from infection. J. Nutr. Biochem..

[26] Vandermosten L., Pham T. T., Possemiers H., Knoops S. (2018). Experimental
malaria-associated acute respiratory distress syndrome is dependent
on the parasite-host combination and coincides with normocyte invasion. Malar. J..

[27] Vieira-Santos F., Brito R. M., Lopes C. A., Fujiwara R. T. (2024). Alveolar
macrophages and monocyte subpopulations during Plasmodium berghei
NK65 experimental malaria-associated acute respiratory distress syndrome. Heliyon.

[28] Taniguchi T., Miyauchi E., Nakamura S., Hirai M. (2015). Plasmodium
berghei ANKA causes intestinal malaria associated with dysbiosis. Sci. Rep..

[29] Van
den Steen P. E., Geurts N., Deroost K., Van Aelst I. (2010). Immunopathology and dexamethasone therapy in a new model for malaria-associated
acute respiratory distress syndrome. Am. J.
Respir. Crit. Care Med..

[30] Godfrey D. G. (1957). Antiparasitic
action of dietary cod liver oil upon Plasmodium berghei and its reversal
by vitamin ″E″. Exp. Parasitol..

[31] Levander O. A., Ager A. L., Morris V. C., May R. G. (1989). Qinghaosu,
dietary vitamin E, selenium, and cod-liver oil: effect on the susceptibility
of mice to the malarial parasite Plasmodium yoelii. Am. J. Clin. Nutr..

[32] Fujikawa M., Kamitani T., Tunru I. S., Yamazaki K. (1993). Hamazaki.T. Antimalarial
effects of purified and alpha-tocopherol-fortified n-3 polyunsaturated
fatty acids. J. Nutr. Biochem..

[33] Hainault I., Carlotti M., Hajduch E., Guichard C., Lavau M. (1993). Fish oil in
a high lard diet prevents obesity, hyperlipidemia, and adipocyte insulin
resistance in rats. Ann. N.Y. Acad. Sci..

[34] Ruzickova J., Rossmeisl M., Prazak T., Flachs P. (2004). Omega-3
PUFA of marine origin limit diet-induced obesity in mice by reducing
cellularity of adipose tissue. Lipids.

[35] Sánchez-Lara K., Turcott J. G., Juárez-Hernández E., Nuñez-Valencia C. (2014). Effects of an oral nutritional
supplement containing eicosapentaenoic acid on nutritional and clinical
outcomes in patients with advanced non-small cell lung cancer: randomised
trial. Clin. Nutr..

[36] Aredes M. A., Camara A. O., De Paula N. S., Fraga K. Y. D. (2019). Efficacy of ω-3 supplementation on nutritional
status, skeletal
muscle, and chemoradiotherapy toxicity in cervical cancer patients:
a randomized, triple-blind, clinical trial conducted in a middle-income
country. Nutrition.

[37] Abdalla S. H. (1988). Peripheral
blood and bone marrow leucocytes in Gambian children with malaria:
numerical changes and evaluation of phagocytosis. Ann. Trop. Paediatr..

[38] Engwerda C. R., Beattie L., Amante F. H. (2005). The importance
of the spleen in malaria. Trends Parasitol..

[39] Casals-Pascual C., Kai O., Cheung J. O., Williams S. (2006). Suppression of erythropoiesis
in malarial anemia is associated with hemozoin in vitro and in vivo. Blood.

[40] Lacerda M. V. G. d., Zackiewicz C., Alecrim W. D., Alecrim M. G. (2007). The neglected Plasmodium
vivax: are researchers from endemic areas really concerned about new
treatment options?. Rev. Soc. Bras. Med. Trop..

[41] De
Azevedo-Quintanilha I. G., Medeiros-de-Moraes I. M., Ferreira A. C., Reis P. A. (2020). Haem oxygenase protects
against thrombocytopaenia and malaria-associated lung injury. Malar. J..

[42] Grau G. E., Piguet P. F., Gretener D., Vesin C., Lambert P. H. (1988). Immunopathology
of thrombocytopenia in experimental malaria. Immunology.

[43] Gupta N. K., Bansal S. B., Jain U. C., Sahare K. (2013). Study of thrombocytopenia
in patients of malaria. Trop. Parasitol..

[44] Dobbs K. R., Crabtree J. N., Dent A. E. (2020). Innate immunity to malaria - the
role of monocytes. Immunol. Rev..

[45] Anstey N. M., Russell B., Yeo T. W., Price R. N. (2009). The pathophysiology
of vivax malaria. Trends Parasitol..

[46] Stevenson M. M., Riley E. M. (2004). Innate immunity
to malaria. Nat.
Rev. Immunol..

[47] Gonçalves R. M., Scopel K. K. G., Bastos M. S., Ferreira M. U. (2012). Cytokine Balance
in Human Malaria: Does Plasmodium Vivax Elicit More Inflammatory Responses
than Plasmodium falciparum?. PLoS One.

[48] Moore K. W., Malefyt R. d. W., Coffman R. L., O’Garra A. (2001). Interleukin-10
and the Interleukin-10 Receptor. Annu. Rev.
Immunol..

[49] Maynard C. L., Weaver C. T. (2008). Diversity in the Contribution of Interleukin-10 to
T-cell-mediated Immune Regulation. Immunol.
Rev..

[50] Joss A., Akdis M., Faith A., Blaser K., Akdis C. A. (2000). IL-10 Directly
Acts on T Cells by Specifically Altering the CD28 Co-Stimulation Pathway. Eur. J. Immunol..

[51] Taylor W. R., Hanson J., Turner G. D., White N. J., Dondorp A. M. (2012). Respiratory
manifestations of malaria. Chest.

[52] Liu C. H., Kuo S. W., Ko W. J., Tsai P. R. (2017). Early
measurement of IL-10 predicts the outcomes of patients with acute
respiratory distress syndrome receiving extracorporeal membrane oxygenation. Sci. Rep..

[53] Mukherjee D., Chora A. F., Lone J. C., Ramiro R. S., Blankenhaus B. (2022). Host lung microbiota promotes malaria-associated
acute respiratory
distress syndrome. Nat. Commun..

[54] D’Avila H., Melo R. C., Parreira G. G., Werneck-Barroso E. (2006). Mycobacterium bovis bacillus Calmette-Guerin
induces TLR2-mediated
formation of lipid bodies: intracellular domains for eicosanoid synthesis
in vivo. J. Immunol..

[55] Ball H. J., MacDougall H. G., McGregor I. S., Hunt N. H. (2004). Cyclooxygenase-2
in the pathogenesis of murine cerebral malaria. J. Infect. Dis..

[56] Bozza P. T., Bakker-Abreu I., Navarro-Xavier R. A., Bandeira-Melo C. (2011). Lipid body
function in eicosanoid synthesis: an update. Prostaglandins, Leukotrienes Essent. Fatty Acids.

[57] Shryock N., McBerry C., Gonzalez R. M. S., Janes S. (2013). Lipoxin
A4 and 15-epi-lipoxin A4 protect against experimental cerebral malaria
by inhibiting IL-12/IFN-γ in the brain. PLoS One.

[58] Sriboonvorakul N., Chotivanich K., Silachamroon U., Phumratanaprapin W. (2023). Intestinal injury and
the gut microbiota in patients with Plasmodium
falciparum malaria. PLoS Pathog..

[59] Robertson R. C., Kaliannan K., Strain C. R., Ross R. P. (2018). Maternal
omega-3 fatty acids regulate offspring obesity through persistent
modulation of gut microbiota. Microbiome.

[60] Meijer K., de Vos P., Priebe M. G. (2010). Butyrate
and other short-chain fatty
acids as modulators of immunity: what relevance for health?. Curr. Opin. Clin. Nutr. Metab. Care.

[61] Saleri R., Borghetti P., Ravanetti F., Cavalli V. (2022). Effects
of different short-chain fatty acids (SCFA) on gene expression of
proteins involved in barrier function in IPEC-J2. Porcine Health Manage..

[62] Valentine C. J., Khan A. Q., Brown A. R., Sands S. A. (2021). Higher-dose
DHA supplementation modulates immune responses in pregnancy and is
associated with decreased preterm birth. Nutrients.

[63] Carlson S. E., Gajewski B. J., Valentine C. J., Kerling E. H. (2021). Higher
dose docosahexaenoic acid supplementation during pregnancy and early
preterm birth: a randomised, double-blind, adaptive-design superiority
trial. Clin. Med..

[64] Munhoz J., Newell M., Bigras G., Goruk S. (2025). Safety
and efficacy of docosahexaenoic acid supplementation during neoadjuvant
breast cancer therapy: Findings from the phase II, double-blind, randomized
controlled DHA-WIN trial. Int. J. Cancer.

