# Supplementary material for: Differential Effects of DHA-Rich Fish Oil Supplementation on Intestinal and Pulmonary Alterations in Experimental Malaria Caused by Plasmodium berghei NK65
Source: ACS Omega. 2026 Mar 20;11(12):19534–46. doi: 10.1021/acsomega.5c12964 (PMC13044828; doi:10.1021/acsomega.5c12964)
Supplement: Supplementary file 1 [file ao5c12964_si_001.pdf]

SUPORTING INFORMATION FOR:

DIFFERENTIAL EFFECTS OF DHA-RICH FISH OIL SUPPLEMENTATION ON INTESTINAL AND PULMONARY ALTERATIONS IN EXPERIMENTAL MALARIA CAUSED BY *Plasmodium berghei* NK65.

Ludmila Ponce Monken Custódio Pereira[1*], José Henrique Silva Rodrigues[1*], Fernanda Martins Pinheiro[1*], Letícia Ferreira Machado[1*], Ana Carolina Fonseca da Silva[1], Carolina Brandi Marques[1*], Milena Gomes Tavares[1*], Fernanda Mikaela Moreira Gonçalves[1], Jéssica Correia Bezerra Bellei[2], Gabriel Brum[3]; Vanessa Cordeiro Dias[1*], João Renato Hipólito[4]; Andre Netto Bastos[5]; Haroldo Lobo dos Santos Nascimento[6], Flávia Marcia de Castro e Silva[7]; Heloisa D'Avila[3]; Adolfo Firmino Neto[6], Janildo Ludolf Reis Junior[6], Vinicius Novaes Rocha[5], Flávia Lima Ribeiro-Gomes[8], Juciane Maria de Andrade Castro[1], Kézia Katiani Gorza Scopel[1*]#

[1*] Research Centre of Parasitology. Department of Parasitology, Microbiology and Immunology and Post-Graduate Program in Biological Science, Federal University of Juiz de Fora, Juiz de Fora, Brazil.

[2] Reproductive Biology Centre. Federal University of Juiz de Fora, Juiz de Fora, Brazil.

[3] Cell Biology Laboratory, Department of Biology, Federal University of Juiz de Fora, Juiz de Fora, Brazil.

[4] Cortes Villela Laboratory, Juiz de Fora, Brazil.

[5] Laboratory of Histology, Department of Morphology, Federal University of Juiz de Fora, Juiz de Fora, Brazil.

[6] Research Centre of Pathology and Veterinary Histology. Department of Veterinary Medicine, Federal University of Juiz de Fora, Juiz de Fora, Brazil.

[7] Department of Parasitology, Microbiology and Immunology, Federal University of Estado do Rio de Janeiro, Brazil.

[8] Laboratory of Malaria Research. Oswaldo Cruz Institute, Fiocruz, Rio de Janeiro, Brazil.

#Corresponding author: Kézia K.G. Scopel
(keziagscopel@gmail.com/kezia.scopel@ufjfj.br)

SUPPLEMENTARY METHODS

Table S1: Nutritional information for the super concentrated DHA oil supplement (Essential Nutrition).

**Servings per container: 30**<br>**Serving size: 4.3g (3 capsules)**		
**Amount per serving**	**4.3g**	**%DV***
Energy (kcal)	27	1
Total fat (g)	3	5
Saturated fat (g)	0	0
Trans fat (g)	0	0
Polyunsaturated fat (g)	2.8	14
Ômega-3 (mg)	1950	49
EPA (mg)	450	-
DHA (mg)	1500	-
Vitamin E (mg)	9	60
Does not contain significant amounts of carbohydrates, total sugars, added sugars, protein, dietary fiber, or sodium.		
*Percent daily values (%DV) are based on a 2000 kcal diet		

Biochemical Tests (TGO and TGP)

To assess liver function, 50 µL of serum collected on the 11th dpi was used to determine TGO (aspartate aminotransferase - AST) and TGP (alanine aminotransferase - ALT) levels using commercial kits. The protocols were performed according to the manufacturer's recommendations (Bioclin).

Isolation and Phenotyping of Splenic Cells

Considering that the spleen is a central lymphoid organ in the immune response to pathogens, the phenotypic characterization of the experimental groups supplemented or not with oil was performed to identify possible changes in the immune profile. To this end, the spleen was collected, weighed, washed with 1X PBS, and placed in a Petri dish containing RPMI 1640 medium with 10% SFB (Sigma-Aldrich). To obtain the cells, the organ was macerated in a cell strainer (70 µm, BD Biosciences) with 5 mL of RPMI medium with 10% SFB, using a syringe plunger. The cell suspensions were centrifuged at 400 g for 10 minutes at 4°C, the supernatant was discarded, and the sediment was resuspended in 5 mL of ACK lysis buffer (0.15 M ammonium chloride, 0.01 M potassium bicarbonate, 0.012 mM EDTA). After 10 minutes at room temperature, the ACK was neutralized with 10 mL of RPMI medium with 10% FBS, and the suspension was centrifuged at 400 g for 5 minutes at 4°C. The supernatant was discarded, and the sediment was resuspended in 5 mL of RPMI with 10% FBS. Viable cells were counted using Trypan Blue, and the cell concentration was adjusted to 10^6 live cells/well in 96-well plates (V-bottom). The plates were then centrifuged at 500 g for 10 minutes at 4 °C. The supernatant was discarded, and the cells were resuspended in 50 µL of cell viability marker (LIVE/DEAD™ Fixable Violet Dead Cell Stain Kit, Invitrogen) with dilutions according to the manufacturer's recommendations and incubated for 30 min at 4°C. Subsequently, the cells were washed with 1X PBS plus 5% SFB and centrifuged again at 500g for 10 min at 4°C, then incubated for 30 min at 4°C with 50 µL of FACS buffer containing the antibodies: anti-mouse CD11b PerCP-Cy5.5 (M1/70, BD Pharmingen); antimouse Ly6C PE-Cy7 (AL-21, BD Pharmingen); antimouse Ly6G APC (1A8; BD Pharmingen); antimouse CD4 APC-H7 (GH1.5, BD Pharmingen); or antimouse CD8 AF700 (53-6.7; BD Pharmingen). After incubation, unbound antibodies were removed by washing, and the cells were resuspended in 200 µL of PBS and analyzed by flow cytometry. Approximately 1000,000 events per sample were acquired using a BD FACSCelesta™, and the data were analyzed using FlowJo software (version 10.0). Cell populations were defined as described: myeloid cells: CD11b+; inflammatory monocytes: CD11b+/Ly6Chi/Ly6-; total T lymphocytes: CD11b-/CD3+; CD4 T lymphocytes: CD3+/CD4+; CD8 T lymphocytes: CD3+/CD8+.

Gate Strategy

A. Lung

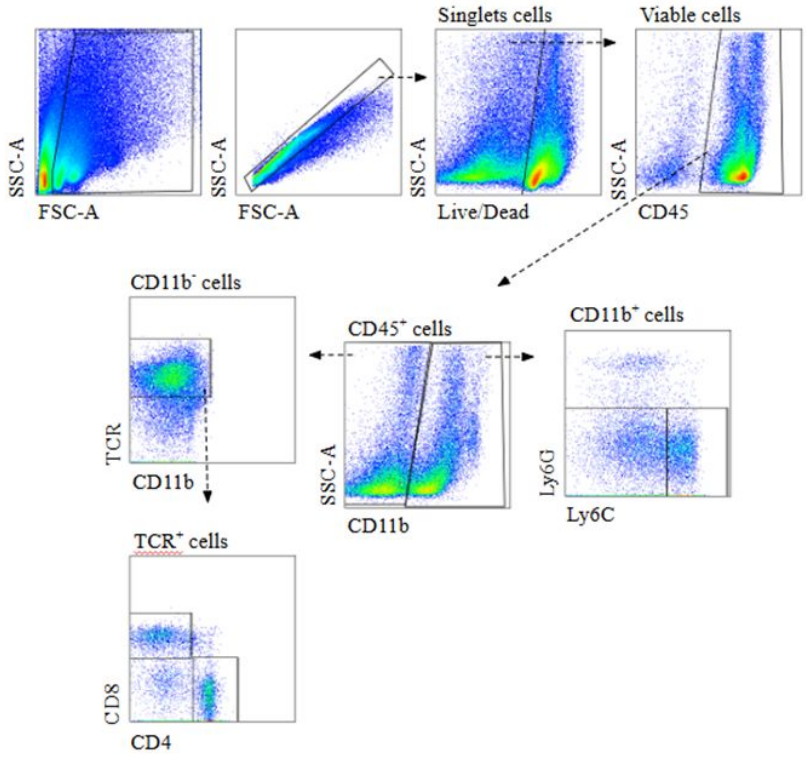

B. Spleen

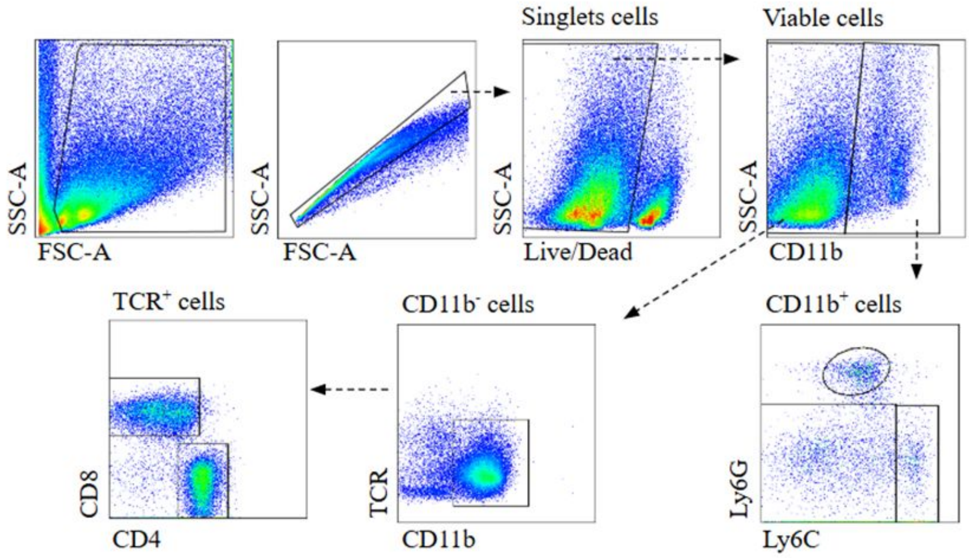

Figure S1: Gate strategy used for immunophenotyping cells in the lungs and spleen of mice supplemented with DHA-rich fish oil and infected with PbN. (A)

Gate strategy used for cells in the lung: CD45+: total leukocytes; CD45+/CD11b+: myeloid cells; CD11b+/Ly6Chi/Ly6-: inflammatory monocytes; CD45+/CD11b-/TCR+: total T lymphocytes; CD11b-/TCR+/CD4+: CD4 T lymphocytes; CD11b-/TCR+/CD8+: CD8 T lymphocytes. (B) Gate strategy used for cells in the spleen: CD11b+: myeloid cells; CD11b+/Ly6Chi/Ly6-: inflammatory monocytes; CD11b-/CD3+: total T lymphocytes; CD3+/CD4+: CD4 T lymphocytes; CD3+/CD8+: CD8 T lymphocytes.

<u>SUPPLEMENTARY RESULTS</u>

Dietary supplementation with DHA does not interfere with parameters such as body tone, trunk curvature, hair erection, dehydration, and incontinence in C57BL/6 mice infected with PbN.

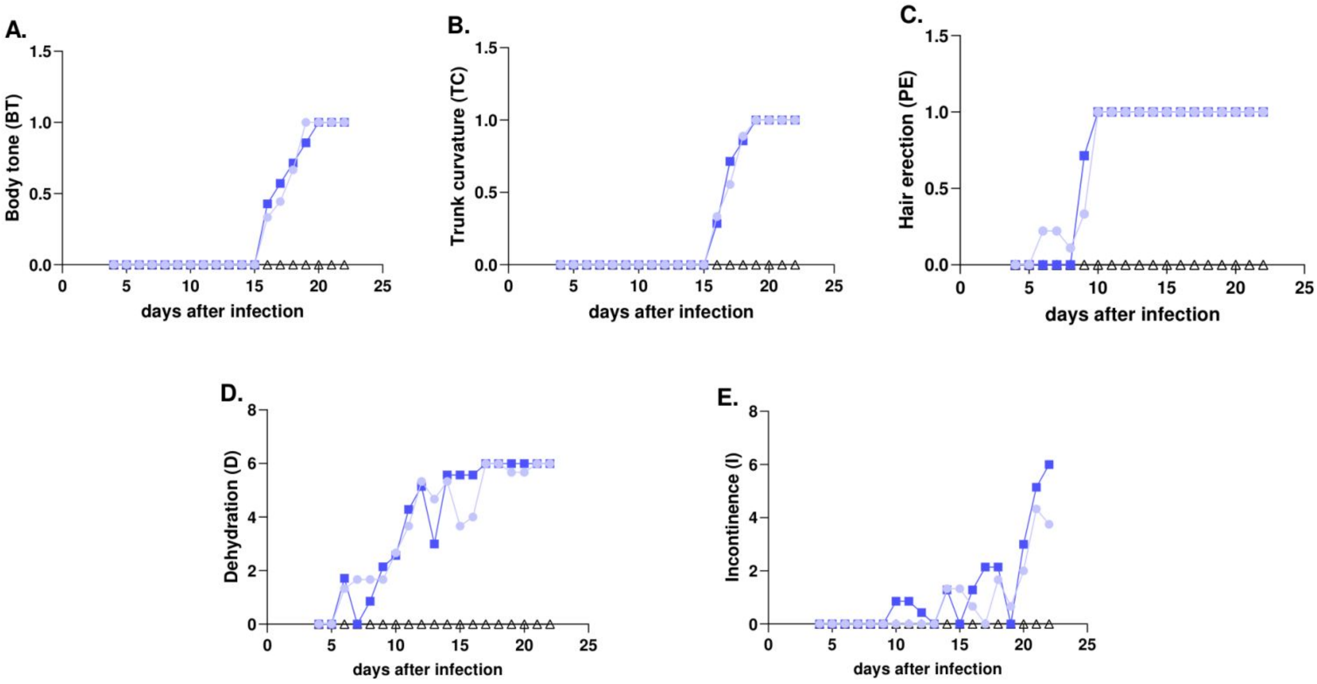

Figure S2: Clinical parameters from mice supplemented with DHA-rich fish oil and infected with PbN. (A) Body tone (BT); (B) Trunk curvature (TC); (C) Hair erection (PE); (D) Dehydration (D); (E) Incontinence (I). Results are shown as frequency or mean ± SEM. CTL: unsupplemented and uninfected mice (triangle); PbN: mice infected with *Plasmodium berghei* NK65 (circle); DHA/PbN: mice supplemented with 3 g/kg DHA and infected with PbN (square).

Dietary supplementation with DHA does not influence TGO and TGP rates in C57BL/6 mice infected with PbN.

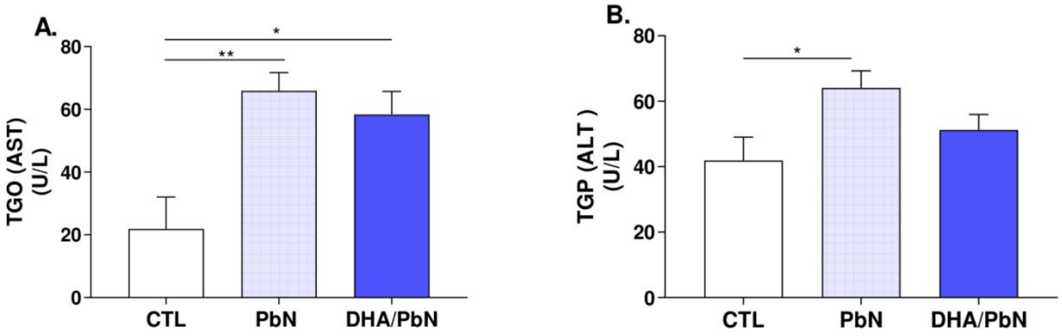

Figure S3: TGO and TGP levels in the serum of mice supplemented with DHA-rich fish oil and infected with PbN. (A) TGO (AST - Aspartate Aminotransferase; (B) TGP (ALT - Alanine Aminotransferase). Results are shown as mean ± SEM. (*) Indicates statistical difference: *p < 0.05; **p < 0.01. CTL: unsupplemented and uninfected mice; PbN: mice infected with *Plasmodium berghei* NK65; DHA/PbN: mice supplemented with 3g/kg of DHA and infected with PbN.

Dietary supplementation with DHA does not alter the profile of cell populations present in the spleen of C57BL/6 mice infected with PbN.

The phenotypic profile of cells present in the spleen tissue of infected animals, supplemented or not with fish oil containing 3g DHA/kg body weight, was analyzed. It was observed that, regardless of the cell population analyzed (myeloid cells, inflammatory monocytes, T lymphocytes, CD4 T lymphocytes, and CD8 T lymphocytes), there was a significant increase in the number of cells in infected animals compared to controls. Interestingly, a tendency toward a reduction in the number of total T lymphocytes, CD4 T lymphocytes, and CD8 T lymphocytes was observed in animals in the DHA/PbN group when compared to those in the PbN group (**Figure S4 A-E**).

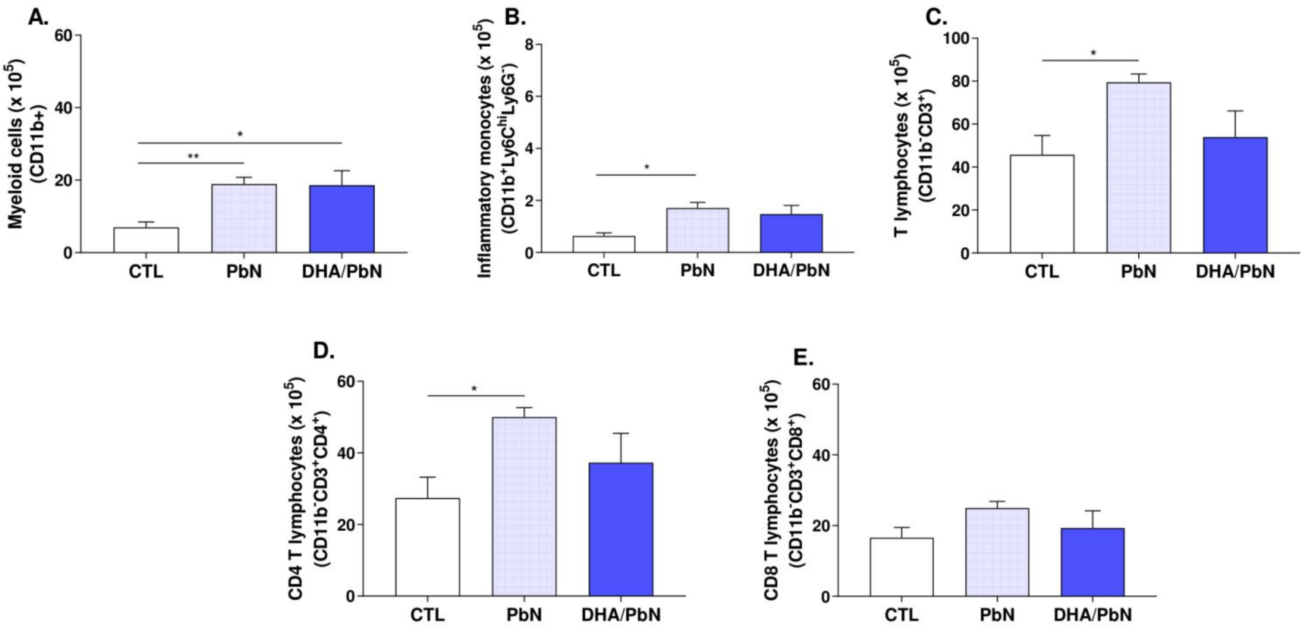

Figure S4: Phenotypic profile of cell populations in the spleen of mice supplemented with DHA-rich fish oil and infected with PbN. (A) Myeloid cells; (B) Inflammatory monocytes; (C) Total T lymphocytes; (D) CD4 T lymphocytes; (E) CD8 T lymphocytes. The graphs show the mean number of cells expressing the specific receptor(s) x 10^5 cells. Results are representative of 3 independent experiments (n=5-6 mice/group) and are shown as mean ± SEM. (*) Indicates statistical difference: *$p < 0.05$; **$p < 0.01$. CTL: unsupplemented and uninfected mice; PbN: mice infected with *Plasmodium berghei* NK65; DHA/PbN: mice supplemented with 3 g/kg of DHA and infected with PbN.
